# Supplementary material for: Effect of intra- and inter-specific plant interactions on the rhizosphere microbiome of a single target plant at different densities
Source: PLoS One. 2025 Jan 27;20(1):e0316676. doi: 10.1371/journal.pone.0316676 (PMC11771940; doi:10.1371/journal.pone.0316676)
Supplement: S1 Table — This table shows the plant count for each of the 21 treatments with the black boarder representing each pot. From these 21 treatments, there were 36 different rhizosphere samples as noted by the labels in bold. (PDF) [file pone.0316676.s001.pdf]

S1 Table. Experimental setup of the increasing plant densities and diversities.

**A1**<br><br>1 Alfalfa Plant	**Ab2/ Ba2**<br><br>1 Alfalfa Plant<br>1 Brassica Plant	**Abf3/ Baf3/ Fab3**<br><br>1 Alfalfa Plants<br>1 Brassica Plants<br>1 Fescue Plants
**A24**<br><br>24 Alfalfa Plants	**Ab24/ Ba24**<br><br>12 Alfalfa Plants<br>12 Brassica Plants	**Abf24/ Baf24/ Fab24**<br><br>8 Alfalfa Plants<br>8 Brassica Plants<br>8 Fescue Plants
**A48**<br><br>48 Alfalfa Plants	**AB48/ Ba48**<br><br>24 Alfalfa Plants<br>24 Brassica Plants	**Abf48/ Baf48/ Fab48**<br><br>16 Alfalfa Plants<br>16 Brassica Plants<br>16 Fescue Plants
**B1**<br><br>1 Brassica Plant	**Bf2/ Fb2**<br><br>1 Brassica Plants<br>1 Fescue Plants	
**B24**<br><br>24 Brassica Plants	**Bf24/ Fb24**<br><br>12 Brassica Plants<br>12 Fescue Plants	
**B48**<br><br>48 Brassica Plants	**Bf48/ Fb48**<br><br>24 Brassica Plants<br>24 Fescue Plants	
**F1**<br><br>1 Fescue Plant	**Fa2/ Af2**<br><br>1 Alfalfa Plants<br>1 Fescue Plants	
**F24**<br><br>24 Fescue Plants	**Fa24/ Af24**<br><br>12 Alfalfa Plants<br>12 Fescue Plants	
**F48**<br><br>48 Fescue Plants	**Fa48/ Af48**<br><br>24 Alfalfa Plants<br>24 Fescue Plants	
