# Supplementary material for: Effect of intra- and inter-specific plant interactions on the rhizosphere microbiome of a single target plant at different densities
Source: PLoS One. 2025 Jan 27;20(1):e0316676. doi: 10.1371/journal.pone.0316676 (PMC11771940; doi:10.1371/journal.pone.0316676)
Supplement: S1 Protocol — (PDF) [file pone.0316676.s002.pdf]

Last updated: 7Jan2022

S1 Protocol. Library preparation and sequencing for MinION – Manter Lab Protocol

Specific Reagents

Phusion Hot Start II Master Mix **(Thermo Scientific, F565L)**
SYBR Green dye I **(Invitrogen S7563)**
ZymoBIOMICS Microbial Community DNA Standard (**Zymo D6306 or D3605)**
AMPure XP Beads or SPRI beads
PCR Barcoding Expansion 1-96 kit **(Nanopore, EXP-PBC096)**
Ligation Sequencing Kit **(Nanopore, SQK-LSK109)**

Primers

Bact_27F-Mn 5' – *TTTCTGTTGGTGCTGATATTGC* AGRGTTYGATYMTGGCTCAG – 3'
Bact_1492R-Mn 5' – *ACTTGCCTGTCGCTCTATCTTC* TACCTTGTTACGACTT – 3'

Notes

Samples amplified in **triplicate reactions for PCR1** and **single reactions for barcoding PCR2**

Step 1: DNA Extraction (See detailed extraction protocol near end of document)

1. Extract 0.25 g field moist soil using Qiagen PowerSoil PRO extraction kit (Qiagen, 47014) and elute in 100 µL C6 buffer using QIAcube.
2. Use a separate 2+ grams of field moist soil sample for determining soil moisture content.

Step 2: PCR1

1. In a new PCR plate, **dilute all DNA extracts 1:20 with nuclease-free H_2O** (e.g., 4 µL DNA, 76 µL H_2O).
2. Dilute P. putida and Zymo DNA Standards to 1 ng/µL in nuclease-free H_2O.
3. Set up PCR1 as follows:

Prepare master mix for triplicate reactions:
Mastermix/Reaction: (X # rxns + extra)

- 10 µL Phusion HSII master mix
- 2.2 µL H_2O
- 0.4 µL forward primer
- 0.4 µL reverse primer
- 5 µL SYBR dye I(20X)

Reagent	µL /rxn	Final Conc.
gDNA (diluted 1:20)	2	-
Phusion Mix	10	1X
dH2O	4.2	-
Primer F (10 µM)	0.4	0.2
Primer R (10 µM)	0.4	0.2
SYBR Green dye (20X)	3	3X
**Total (µL)**	**20**	

Mix & Aliquot **18 µL Mastermix per well**
Add 2 µL template: gDNA (diluted 1:20), ZymoBIOMICS standard (1 ng/µL), or H_2O to well for **20 µl total/reaction**
Prepare standard series: Four steps of 1:10 dilutions, starting with 2 µL of 1ng/µL of P. putida stock

Thermocycle for 25 cycles using protocol →→→→→

16S.minION.PCR1			
1. 98C for 30 sec			
2. 25X (98C for 15 sec, 50C for 15 sec, 72C for 60 sec)			
3. 72C for 5:00			

Step 3: Pooling of triplicate PCR1 rxn

1. After PCR1, combine replicates by mixing equal volumes (15 µL) of each triplicate rxn into a new PCR plate and proceed to bead purification.

Last updated: 7Jan2022

Step 4: Bead Purification (detailed protocols below) – Purify 45 uL PCR1

1. Bring AMPure XP beads (same volume as PCR sample) and PCR samples to room temperature (this is critical).
2. Make fresh (this is critical) 70% EtOH, enough for 200 µL per sample. Dilute based on volumetric part, not by bringing to volume (i.e. 70 mL absolute EtOH + 30 mL nuclease-free water).
3. Bead clean using your method of choice. Key steps are to use a 1:1 (v:v) bead to PCR ratio and a final elution volume of 40 µL nuclease-free H_2O.

Optional Step 5: Visualize 5 µL of purified product on 0.8% gel with 1 kb ladder

Estimate amplicon concentration from the band intensity compared to the ladder

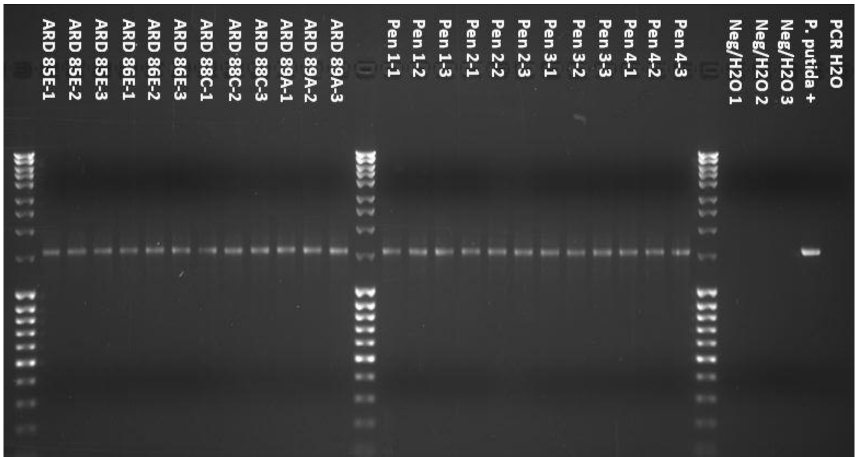

Bead cleaned PCR1 products, 1% Agarose gel
ThermoFisher MassRuler DNA Ladder Mix

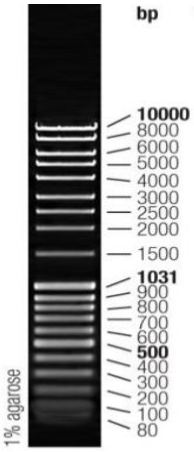

Step 6: Qubit

We do not use this info for modification.

Step 7: PCR2 (barcoding)

1. In a new PCR plate, **dilute all PCR1 products 1:10 with nuclease-free H_2O** (e.g., 4 µL PCR1, 36 µL H_2O)
2. Set up PCR1 as follows:

Prepare Phusion/H2O master mix for reactions (single reaction per sample):

Mastermix/Reaction: (X # rxns + extra)

- 25 µL Phusion HSII master mix
- 19 µL H_2O

Mix & Aliquot **44 µL Mastermix per well**

Add to each well:

- 1 µL sample-specific barcode primer*
- 5 µL purified 1:10 diluted PCR product or H_2O for negative control

Reagent	µL /rxn	Final Conc.
PCR1 product (diluted 1:10)	5	-
Phusion Mix	25	1X
dH2O	19	-
Barcode	1	-
**Total (µL)**	**50**	

*Barcodes are numbered BC01-BC096, <u>*do not*</u> *use Barcode Adapters (BCA01-96)*

Thermocycle for 15 cycles using protocol →→→→→

16S.minION.PCR2
1. 98C for 30 sec
2. 15X (98C for 15 sec, 62C for 15 sec, 72C for 60 sec)
3. 72C for 5:00

Last updated: 7Jan2022

Optional Step 8: Visualize 5 µL PCR product on a 0.8% gel with 1 kb ladder

Load samples on gel. Include one non-barcoded PCR product to show an upward shift in length from barcode addition. Verify amplification of product and lack of bands in negative controls.

Step 9: Bead purification

1:1 PCR2 product:Beads
Elute in 40 µL H_2O

Optional: Qubit (If you have different primers or amplicon lengths)

Step 10: Pool at equal volumetric/molar ratios

If single amplicon length, pool 5 µL from each PCR2 reaction.
If multiple amplicon lengths, pool based on equal molarity (~0.5 nM).
Final pooled DNA should be 8-10 ng/µL or 0.5 nM.

Step 11: Qubit final pool, nanodrop if possible for quality

Prepare the barcoded library for MinION sequencing:

Consumables:

- Pooled bead-cleaned PCR2 DNA (~400 ng or 50 µL @ 8-10 ng / µL)
- Ligation sequencing kit (Nanopore SQK-LSK109; enough for 6 uses)
  (SQT and DCS reagents will not be used)(LNB is limiting reagent)
- Flow cell priming kit (Nanopore EXP-FLP001)
- NEBNext End repair / dA-tailing Module (E7546)
- NEB Quick T4 DNA Ligase
- Agencourt AMPure XP beads
- Fresh 70% ethanol in nuclease-free water
- 0.2ml thin walled PCR tubes or PCR plate
- 1.5ml Eppendorf low bind tubes
- Nuclease-free water

Prepare Library

Dilute pooled PCR2 bead-purified DNA as above to (~400 ng or 50 µL @ 8-10 ng / µL)

Before starting each of the following protocol parts, the listed reagents should be thawed at room temperature, mixed, spun down, and placed on ice.

End-repair/dA-tailing *(Reagents: Ultra II End-prep buffer, Ultra II End-prep enzyme mix)*

1. In a 0.2mL PCR tube or 96 well PCR plate, add the following:
   - 50 µL Pooled bead-cleaned PCR2 DNA (8-10 ng/ µL)
   - 7 µL Ultra II End-prep reaction buffer
   - 3 µL Ultra II End-prep enzyme mix
   - Optional: 1 ul DCS (for downstream troubleshooting)
2. Mix gently by flicking the tube and then spin down.
3. Using a thermal cycler, incubate at 20°C for 5 minutes and 65°C for 5 min.
   *During wait period, start prepping beads/ethanol for next steps.

Last updated: 7Jan2022

<u>Bead Clean #1</u> *(Reagents: SPRI or AMPure XP beads, 70% Ethanol)*

1. Prepare the AMPure XP beads; resuspend by vortexing, bring to room temp. You will need 60 µL for this step, and another 40 µL later on.
2. Prepare 500 µL of fresh 70% ethanol in Nuclease-free water.
3. Transfer your 60 µL of end-prepped DNA to a 1.5 mL tube.
4. Add 60 µL of resuspended beads to the DNA and mix by flicking.
5. Incubate on rotator mixer for 5 minutes at room temp.
6. Spin down sample and pellet beads on a magnet. Keep the tube on magnet and carefully pipette off supernatant. Discard supernatant.
7. Keep on magnet, wash beads with 200 µL freshly prepared 70% ethanol without disturbing the pellet. Wait ~30 seconds, remove ethanol using a pipette and discard.
8. Repeat the previous step.
9. Remove the tube from the magnetic rack and resuspend the pellet in 61 µL Nuclease-free water. Incubate for 2 minutes at room temp.
10. Place tube back on magnet, allow beads to pellet.
11. Remove and retain 61 µL of eluate into a new 1.5 mL tube. Do not transfer beads.

<u>Adapter Ligation</u> *(Reagents: LNB, T4 Ligase, AMX)*

1. In a 1.5 mL tube, mix in the following order:
   - 60 µL DNA sample from previous step
   - 25 µL LNB
   - 10 µL T4 Ligase
   - 5 µL AMX
2. Mix gently by flicking the tube, spin down.
3. Incubate for 10 minutes at room temp.

<u>Bead Clean #2</u> *(Reagents: SPRI or AMPure XP beads, SFB, EB)*

1. Add 40 µL resuspended AMPure XP beads to the reaction. Mix by flicking.
2. Incubate on rotator mixer for 5 minutes at room temp.
3. Spin down sample and pellet beads on a magnet. Keep the tube on magnet and carefully pipette off supernatant. Discard supernatant.
4. Wash the beads by adding 250 µL SFB. Flick the beads to resuspend, then return the tube to the magnet and allow the beads to pellet. Remove the supernatant using a pipette and discard.
5. Repeat the previous step.
6. Spin down and place tube back on magnet. Pipette off any SFB. Allow to dry for ~30 seconds. Do not over-dry the pellet to the point of cracking.
7. Remove the tube from the magnetic rack and resuspend the pellet in 15 µL EB.
8. Incubate for 10 minutes at room temp. (You may want to start priming the flow cell during this period)
9. Place tube back on magnet, allow beads to pellet.
10. Remove and retain 15 µL of eluate into a new 1.5 mL tube. Do not transfer beads.

<u>Quantify</u>
It is recommended to load 5-50 femtomoles (fmol) of DNA library to the flow cell. Follow these steps to figure out what volume of library to add.

1. Using Qubit fluorometer, quantify 1 µL of eluted sample.
2. Use dsDNA mass to moles convertor: https://nebiocalculator.neb.com/#!/dsdnaamt to calculate your femtomoles per µL of library.

Last updated: 7Jan2022
EXAMPLE: My Qubit value is 9.3 ng/µL. This converts to 10.03 fmol/µL for a 1.5kb DNA fragment. I used 5 µL of my library plus 7 µL of H_2O for the final prep solution, result is 50.15 fmol of DNA to be added to the flow cell.

<u>Priming and loading the flow cell</u> *(Reagents: FLT, FB, SQB, LB)*

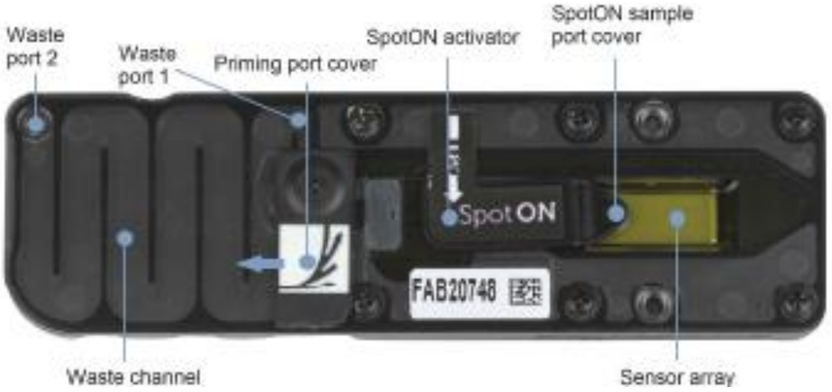

1. Perform a flow-cell check:
    a. Insert the flow-cell into the sequencer and connect the sequencer to the computer. If the flow cell is being reused, ensure it is loaded with storage buffer (SB) from the wash kit.
    b. Open MinKNOW and wait for the device to be recognized.
    c. Select the flow cell type (#106 for the starter kit).
    d. Then select "check flow cells" at the bottom of the screen.
    e. Monitor progress by selecting the blue drop-down button and selecting "current experiment"
    f. Once the check is complete, hover the mouse over the green or yellow icon at the top right of the flow cell picture. The number of available pores will appear. Aim for >800 pores.
2. Open the lid of the nanopore sequencing device and slide the flow cell's priming port cover clockwise so that the priming port is visible.
3. Check for small bubble under the priming port cover, to remove bubble:
    a. Set a P1000 pipette to 200 µL
    b. Insert the tip into the priming port
    c. Slowly turn the pipette wheel until the dial shows 220-230 µL, or until you can see a small volume of buffer entering the pipette tip.
4. Prepare the flow cell priming mix:
    a. Add 30 µL of thawed and freshly mixed FLT directly to the tube of thawed and mixed FB
    b. Mix by pipetting up and down
5. Load 800 µL of the priming mix into the flow cell via the priming port, avoiding the introduction of air bubbles. Wait 5 minutes
6. In a new 1.5 mL tube, prepare the library for loading as follows:
    - 37.5 µL SQB
    - 25.5 µL LB, mixed immediately before use
    - 12 µL DNA library (50 fmol, diluted with H_2O based on above calculation)
    - Complete flow cell priming:
    a. Gently lift the SpotON sample port cover to make the sample port accessible.
    b. Load another 200 µL of the priming mix (FLT+FB) into the flow cell via the priming port (not the sample port). Avoid introduction of air bubbles
7. Mix the prepared library gently by pipetting up and down immediately prior to loading.
8. Add 75 µL of sample to the flow cell via the SpotON sample port in a dropwise fashion. Ensure each drop flows into the port before adding the next.
   If the sample does not enter flow cell easily, double check that priming port cover is OPEN
9. Gently replace the SpotON sample port cover, making sure the bung enters the SpotON sample port.
10. Move the priming port cover back over the priming port. Close the MinION lid.

Last updated: 7Jan2022

Starting the sequencing run:

1. Open MinKNOW program, use USB cord to connect to sequencing device.
2. When device is recognized by the program, select the flow cell type (#106 for the starter kit).
3. Check the “Available” box. Click “New Experiment”.
4. The experiment must be named before you can move on to the next settings.
5. Choose the kit: We are using SQK-LSK109
6. The parameters we have been using are:
    - Basecalling – OFF
    - Runtime – 48 hours
    - Reads per file – 4000
    - Voltage – -180 V
7. Choose “Start Run”.
8. Once the sequencer has warmed up and started the run, ensure that ~65% of available channels (green) are actively reading (light green). This should occur within 15 minutes of beginning sequencing. Low to no channels actively reading suggests and error in library preparation.

Washing and storage

After your sequencing run is complete, if you would like to reuse the flow cell, follow the Wash Kit instructions and store the washed flow cell at 2-8°C. The Wash kit protocol is at:
OneDrive - USDA\MinION\Protocols\FlowCellWashKit_WFC_9088_v1_revB_18Sep2019-any.pdf.

If the flow cell will not be used further, flush and package the flow cell for return using this protocol:
OneDrive - USDA\MinION\Protocols\Device-flow-cell-returns-FRI_S1002_v1_revN_06Apr2016.pdf

Detailed Protocols

QIAcube PowerSoil Pro Extraction Protocol

1. In DNA Extraction Log, record date, tech initials, sample names, and method.
2. Add **800 uL of CD1 solution** to the PowerBead Tube, recap, and vortex briefly.
3. Place tubes in Vortex Genie and run at max speed for 10 minutes.
4. During this step, began preparing the QIAcube and rotor adapters; see next set of instructions.
5. Remove samples from vortex, centrifuge at **15000 rcf for 1 minute**.
6. Carefully **transfer 600uL (at least 450uL) of supernatant to position 2** (middle position) of rotor adapter. Avoid transferring the “pellet”.
7. Place rotor adapter into corresponding number position of QIAcube centrifuge.
8. In QIAcube menu, choose DNA > Powersoil Pro > Soil > IRT > Start
9. When run is complete, remove eluted DNA samples, cap reagent bottles, trash waste tips and tubes, close machine cover and turn off.

Prepare QIAcube and rotor adapters:

1. Place rotor adapters into rotor adapter holder. One per sample in the run.
2. Load MB spin column into position 1 of rotor adapter.
3. Load labeled 1.5mL tube into position 3 of rotor adapter.
4. Add 1000uL tips to QIAcube.
5. Add 2mL round bottom tubes to QIAcube shaker plate.
6. Check and refill reagents, make sure reagent caps are off.

Last updated: 7Jan2022

Bead Cleaning:

Bead cleaning #2 for flow cell loading is different than the protocols below.

Bead clean using your method of choice. Key steps are to use a 1:1 (v:v) bead to PCR ratio and a final elution volume of 40 µL nuclease-free H_2O. There is no limit to binding capacity of beads but modifying the ratio will exclude size fractions

96-well pin magnet (V & P Scientific, Inc.)

1. Bring AMPure XP beads (same volume as PCR sample) and PCR samples to room temperature (this is critical).
2. Make fresh (this is critical) 70% EtOH, enough for 200 µL per sample. Dilute based on volumetric part, not by bringing to volume (i.e. 70 mL absolute EtOH + 30 mL nuclease-free water).
3. Make sure magnetic cover plate is clean (bleach, UV irradiation). Fit onto Magnetic Bead Extractor so there is not a gap between magnetic pins and end of the cover plate's pointed wells.
4. Prepare four 96-well plates: 1 for mixing beads and PCR product, 2 for EtOH washing, and 1 for DNA elution. Plates used for bead mixing and EtOH washing can be reused, if cleaned and sterilized with 10% bleach and UV irradiation.
5. Add 1.0x of AMPure XP beads magnetic beads to each well/tube (e.g. 40 µL beads + 40 µL PCR product). Note: to target longer amplicons, use a lower ratio of beads such as 0.8x. To target maximum DNA recovery regardless of size, use a higher ratio of beads such as 1.2x.
6. Add PCR reaction to beads. Pipette up and down 10 times to mix.
7. Incubate at room temp for 5 minutes.
8. Insert magnetic pins with cover plate attached and allow beads to bind for 1 minute. Note: Try to concentrate beads at the tip (bottom) of the plate as much as possible for maximum elution yield
9. Transfer magnet with beads to new plate with 100 µL 70% EtOH in each well for 30 seconds. Do not remove magnet.
10. Transfer magnet with beads to second plate with 100 µL 70% EtOH per well for 30 seconds. Do not remove magnet.
11. Remove magnet and allow to air-dry for up to 5 minutes. Do not over dry.
12. Release plate cover with attached beads from the magnet into final elution plate with 40 µL water per well. Swirl to release beads and incubate 2 minutes. Add magnet back to cover, remove beads, and discard.

Microcentrifuge tubes

1. Bring AMPure XP beads (same volume as PCR sample) and PCR samples to room temperature (this is critical).
2. Make fresh (this is critical) 70% EtOH, enough for 200 µL per sample. Dilute based on volumetric part, not by bringing to volume (i.e. 70 mL absolute EtOH + 30 mL nuclease-free water).
3. Add 1.0x of AMPure XP beads magnetic beads to each well/tube (e.g. 40 µL beads + 40 µL PCR product). Note: to target longer amplicons, use a lower ratio of beads such as 0.8x. To target maximum DNA recovery regardless of size, use a higher ratio of beads such as 1.2x.
4. Add PCR reaction to beads. Pipette up and down 10 times to mix (do not vortex)
5. Incubate at room temp for 5 minutes.
6. Place tube on magnetic rack and allow beads to bind for 1 minute.
7. Keeping tube on magnet, pipette off supernatant (retain just in case)
8. Keeping tube on magnet, wash with 1× freshly prepared 70% EtOH without disturbing pellet
9. Remove EtOH by pipet and discard
10. Repeat EtOH wash
11. Close tube and leave on magnet for 2 min – remove any residual EtOH
12. Briefly leave at RT or on 37°C thermocycler with tube open to evaporate remaining EtOH *Do not dry pellet
13. Resuspend in H_2O, mix by pipetting, incubate 2 min at RT
14. Transfer supernatant to new tube (discard bead tube)

Last updated: 7Jan2022

Gel electrophoresis:

Large gel box (dimension), in-gel staining

1. Add 400mL TBE 0.5X to 500mL Erlenmeyer flask
2. Mix 4.0 grams SeaKem LE Agarose into flask
3. Microwave until Agarose is fully dissolved (~3 minutes), solution will be clear
4. When flask is cool enough to hold in bare hand, add 20 µL Ethidium Bromide (or other DNA stain), swirl to mix
5. Pour agarose into gel tray with well comb in place and allow gel to set
6. Remove well comb, fill gel box with TBE 0.5× buffer, covering gel
7. Mix 5 µL sample with 1 µL dye before adding to wells. Add 5 µL DNA Ladder to select wells.
8. Run gel at 150V for 2 hours.

Midi gel box (gel dimension 10 cm × 14 cm (L×W)), post staining

1. Add 0.6 g low melt agarose to 250 mL Erlenmeyer flask with 75 ml 1× TBE buffer
2. Microwave for 30 s, swirl to mix and repeat 2 times or until fully dissolved (be careful, agarose can flash)
3. When flask is cool enough to hold in bare hand, pour into gel tray with well combs in place, allow gel to set
4. Transfer to gel box, remove gel combs, fill gel box with 1 × TBE buffer, covering gel
5. Mix 5 µL of PCR with 1 µL 6× loading buffer. Load into wells. Load 1 kb ladders
6. Run gel at 120V for 35 min or until loading buffer is approximately 2/3 way through gel
7. Transfer gel to staining box with Gel Red, Ethidium or Sybr-Safe. Stain/destain according to manufacturer.

Primers (desalted, IDTDNA)

Target (size)	Name/Sequence (Nanopore adapters are bold/underlined)		Ref.	Anneal T
Bacteria (1.5 Kb)	Bact-27F-Mn	(**TTTCTGTTGGTGCTGATATTGC**AGRGTTYGATYMTGGCTCAG)	[1]	50C
	Univ-1492R-Mn	(**ACTTGCCTGTCGCTCTATCTTC**TACCTTGTTACGACTT)	[2] (GM4R)	
Fungi (~2.5 Kb)*	SSU515Fngs-Mn	(**TTTCTGTTGGTGCTGATATTGC**GCCAGCAACCGCGGTAA)	[3, 4]	63C
	TW13-Mn	(**ACTTGCCTGTCGCTCTATCTTC**GGTCCGTGTTTCAAGACG)	[3]	

Specificities: 27F bacteria; 1492R universal; SSU515Fngs most organisms; TW13 eukaryotes;
Specificities for some of the primers are also in [7].
* amplifies rRNA gene and ITS region from V4 of SSU to D2 of LSU

References

1. Lane, D.J., *16S/23S rRNA sequencing*, in *Nucleic acid techniques in bacterial systematics*, E. Stackebrandt and M. Goodfellow, Editors. 1991, John Wiley & Sons Ltd.: West Sussex, United Kingdom.
2. Muyzer, G., et al., *Phylogenetic relationships ofThiomicrospira species and their identification in deep-sea hydrothermal vent samples by denaturing gradient gel electrophoresis of 16S rDNA fragments.* Archives of Microbiology, 1995. **164**(3): p. 165-172.
3. Tedersoo, L., A. Tooming-Klunderud, and S. Anslan, *PacBio metabarcoding of Fungi and other eukaryotes: errors, biases and perspectives.* New Phytol, 2018. **217**(3): p. 1370-1385.
4. Tedersoo, L., et al., *Shotgun metagenomes and multiple primer pair-barcode combinations of amplicons reveal biases in metabarcoding analyses of fungi.* MycoKeys, 2015(10): p. 1-43.
5. DeLong, E.F., *Archaea in coastal marine environments.* Proceedings of the National Academy of Sciences, 1992. **89**(12): p. 5685-5689.
6. Kolganova, T., B. Kuznetsov, and T. Tourova, *Designing and testing oligonucleotide primers for amplification and sequencing of archaeal 16S rRNA genes.* Microbiology, 2002. **71**(2): p. 243-246.
7. Klindworth, A., et al., *Evaluation of general 16S ribosomal RNA gene PCR primers for classical and next-generation sequencing-based diversity studies.* Nucleic acids research, 2013. **41**(1): p. e1-e1.
