# Supplementary material for: Effect of intra- and inter-specific plant interactions on the rhizosphere microbiome of a single target plant at different densities
Source: PLoS One. 2025 Jan 27;20(1):e0316676. doi: 10.1371/journal.pone.0316676 (PMC11771940; doi:10.1371/journal.pone.0316676)
Supplement: S2 Table — Enriched column shows which treatment the bacterial taxa is enriched (A1: single alfalfa plant, A24: 24 alfalfa plants, A48: 48 alfalfa plants). Bacterial taxa which were enriched in only one treatment of increasing plant density is highlighted in orange. Bacterial taxa which were enriched all density treatment is highlighted in sky blue. (PDF) [file pone.0316676.s003.pdf]

S2 Table. Differential abundance comparison of alfalfa when grown alone (1 plant) and alfalfa plant densities.

A24

Bacterial Taxa	Enriched	Log Fold	P-adjust
Larkinella insperata	A1	-20.12	1.28E-03
Leptolyngbya sp. O-77	A1	-18.44	2.44E-03
Halomicronema hongdechloris	A1	-19.20	2.52E-03
Anabaena cylindrica	A1	-18.15	2.52E-03
Telluribacter humicola	A1	-18.03	2.52E-03
Adhaeribacter aerophilus	A24	12.61	2.52E-03
Arthrobacter sp. KBS0702	A24	9.46	5.63E-06
Arthrobacter sp. UKPF54-2	A24	9.16	3.68E-03
Arthrobacter sp. QXT-31	A24	5.15	7.57E-03
Adhaeribacter swui	A24	17.11	7.43E-18
Pseudarthrobacter sp. NIBRBAC000502771	A24	18.79	4.51E-16
Pseudarthrobacter phenanthrenivorans	A24	19.83	6.73E-22

A48

Bacterial Taxa	Enriched	Log Fold	P-adjust
Ammoniphilus oxalaticus	A1	-29.96	2.79E-24
Oscillatoria nigro-viridis	A1	-23.81	2.36E-12
Larkinella harenae	A1	-25.78	2.18E-11
Paenibacillus sp. 37	A1	-20.93	2.19E-10
Azospirillum brasilense	A1	-23.21	2.19E-10
Paenibacillus xylanexedens	A1	-21.14	2.00E-09
Larkinella rosea	A1	-24.28	6.07E-09
Adhaeribacter swui	A48	16.43	3.61E-15
Pseudarthrobacter sp. NIBRBAC000502771	A48	19.10	3.61E-15
Pseudarthrobacter phenanthrenivorans	A48	19.80	4.04E-20

Enriched column shows which treatment the bacterial taxa is enriched (A1: single alfalfa plant, A24: 24 alfalfa plants, A48: 48 alfalfa plants). Bacterial taxa which were enriched in only one treatment of increasing plant density is highlighted in orange. Bacterial taxa which were enriched all density treatment is highlighted in sky blue.
