# Supplementary material for: Effect of intra- and inter-specific plant interactions on the rhizosphere microbiome of a single target plant at different densities
Source: PLoS One. 2025 Jan 27;20(1):e0316676. doi: 10.1371/journal.pone.0316676 (PMC11771940; doi:10.1371/journal.pone.0316676)
Supplement: S3 Table — Enriched column shows which treatment the bacterial taxa is enriched (A1: single alfalfa plant, Ab2: single alfalfa and brassica plant, Ab24: 12 alfalfa and brassica plants, Ab48: 24 alfalfa and brassica plants). Bacterial taxa which were enriched when alfalfa was grown alone as compared to multiple density treatments. Bacterial taxa which were enriched in only one treatment of increasing plant density is highlighted in orange. Bacterial taxa which were enriched in more than one diversity treatment is highlighted in light sky blue. Bacterial taxa which were enriched all density treatment is highlighted in sky blue. (PDF) [file pone.0316676.s004.pdf]

S3 Table. Differential abundance comparison of alfalfa when grown alone (1 plant) and alfalfa-brassica mixtures.

Ab2

Bacterial Taxa	Enriched	Log Fold	P-adjust
Dyadobacter sediminis	A1	-19.86	4.05E-09
Ensifer adhaerens	A1	-20.52	1.40E-04
Exiguobacterium aurantiacum	A1	-17.87	8.40E-08
Exiguobacterium mexicanum	A1	-29.94	4.45E-43
Larkinella harenae	A1	-28.32	1.43E-15
Larkinella rosea	A1	-24.96	1.82E-10
Lysobacter helvus	A1	-21.48	1.77E-03
Noviherbaspirillum suwonense	A1	-7.31	3.49E-04
Peribacillus simplex	A1	-16.77	8.34E-03
Pontibacter chitinilyticus	A1	-30.00	7.74E-30
Pontibacter rhizosphera	A1	-16.65	8.81E-05
Pseudomonas stutzeri	A1	-17.05	3.27E-03
Sinorhizobium fredii	A1	-18.50	1.95E-06
Paucimonas lemoignei	Ab2	17.31	8.34E-03
Gemmata massiliana	Ab2	15.96	8.01E-03
Adhaeribacter swui	Ab2	18.52	2.16E-21

Ab24

Bacterial Taxa	Enriched	Log Fold	P-adjust
Bacillus carboniphilus	A1	-26.49	2.35E-44
Bacillus mannanilyticus	A1	-6.39	6.33E-03
Devosia geojensis	A1	-22.10	5.95E-16
Azohydromonas australica	A1	-25.49	1.60E-03
Trichocoleus desertorum	A1	-21.31	1.61E-05
Gemmata sp. SH-PL17	Ab24	17.99	6.27E-03
Metabacillus indicus	Ab24	2.91	1.74E-03
Arthrobacter sp. KBS0702	Ab24	8.23	1.36E-04
Gemmata massiliana	Ab24	19.13	5.65E-04
Pseudarthrobacter sp. NIBRBAC000502771	Ab24	18.39	1.29E-15
Pseudarthrobacter phenanthrenivorans	Ab24	20.86	1.02E-24
Adhaeribacter swui	Ab24	17.54	4.30E-19

Ab48

Bacterial Taxa	Enriched	Log Fold	P-adjust
Achromobacter insolitus	A1	-29.96	1.32E-31
Luteolibacter pohnpeiensis	A1	-21.51	3.72E-07
Planomicrobium chinense	A1	-22.23	2.30E-09
Stenotrophomonas sp. MYb57	A1	-9.74	7.78E-03
Larkinella insperata	A1	-21.46	1.20E-04
Leptolyngbya sp. O-77	A1	-19.67	2.77E-04
Anabaena cylindrica	A1	-19.67	3.32E-04
Azohydromonas australica	A1	-26.41	4.47E-04
Trichocoleus desertorum	A1	-21.58	7.63E-06
Adhaeribacter aerophilus	Ab48	15.14	3.92E-05
Arthrobacter sp. KBS0702	Ab48	8.09	1.20E-04
Pseudarthrobacter sp. NIBRBAC000502771	Ab48	18.04	5.26E-15
Pseudarthrobacter phenanthrenivorans	Ab48	19.21	6.43E-21
Adhaeribacter swui	Ab48	17.16	2.44E-18

Enriched column shows which treatment the bacterial taxa is enriched (A1: single alfalfa plant, Ab2: single alfalfa and brassica plant, Ab24: 12 alfalfa and brassica plants, Ab48: 24 alfalfa and brassica plants). Bacterial taxa which were enriched when alfalfa was grown alone as compared to multiple density treatments. Bacterial taxa which were enriched in only one treatment of increasing plant density is highlighted in orange. Bacterial taxa which were enriched in more than one diversity treatment is highlighted in light sky blue. Bacterial taxa which were enriched all density treatment is highlighted in sky blue.
