# Supplementary material for: Effect of intra- and inter-specific plant interactions on the rhizosphere microbiome of a single target plant at different densities
Source: PLoS One. 2025 Jan 27;20(1):e0316676. doi: 10.1371/journal.pone.0316676 (PMC11771940; doi:10.1371/journal.pone.0316676)
Supplement: S4 Table — Enriched column shows which treatment the bacterial taxa is enriched (A1: single alfalfa plant, Af2: single alfalfa and fescue plant, Af24: 12 alfalfa and fescue plants, Af48: 24 alfalfa and fescue plants). Bacterial taxa which were enriched when alfalfa was grown alone as compared to multiple density treatments. Bacterial taxa which were enriched in only one treatment of increasing plant density is highlighted in orange. Bacterial taxa which were enriched in more than one diversity treatment is highlighted in light sky blue. Bacterial taxa which were enriched all density treatment is highlighted in sky blue. (PDF) [file pone.0316676.s005.pdf]

S4 Table. Differential abundance comparison of alfalfa when grown alone (1 plant) and alfalfa-fescue mixtures.

Af2

Bacterial Taxa	Enriched	Log Fold	P-adjust
Achromobacter insolitus	A1	-25.05	3.76E-21
Arthrobacter sp. Rue61a	A1	-17.25	7.55E-11
Azospirillum brasilense	A1	-22.15	3.85E-10
Azospirillum sp. TSH58	A1	-19.02	2.10E-04
Dyadobacter sediminis	A1	-19.09	4.62E-08
Larkinella rosea	A1	-23.79	4.13E-09
Luteolibacter pohnpeiensis	A1	-19.70	1.39E-05
Pontibacter rhizosphera	A1	-16.04	4.74E-04
Sphingoaurantiacus polygranulatus	A1	-21.37	3.34E-12
Paenibacillus sp. 37	A1	-19.93	3.85E-10
Paenibacillus xylanexedens	A1	-19.51	1.48E-08
Planomicrobium chinense	A1	-20.21	2.40E-07
Adhaeribacter swui	Af2	15.50	9.61E-14
Pseudarthrobacter sp. NIBRBAC000502771	Af2	17.93	8.12E-14

Af24

Bacterial Taxa	Enriched	Log Fold	P-adjust
Leptolyngbya sp. O-77	A1	-19.09	2.64E-03
Lysobacter helvus	A1	-21.19	6.71E-03
Methylophilus sp. TWE2	A1	-20.97	1.04E-03
Pseudomonas stutzeri	A1	-18.31	3.87E-03
Roseimicrobium gellanilyticum	A1	-20.03	3.31E-06
Telluribacter humicola	A1	-18.03	5.48E-03
Azospirillum sp. TSA2s	A1	-16.89	8.24E-03
Exiguobacterium acetylicum	A1	-21.95	1.10E-10
Exiguobacterium sp. U13-1	A1	-21.99	1.10E-10
Adhaeribacter aerophilus	Af24	15.77	9.40E-05
Arthrobacter sp. KBS0702	Af24	7.65	1.57E-03
Pseudarthrobacter phenanthrenivorans	Af24	17.83	5.07E-16
Adhaeribacter swui	Af24	17.16	5.07E-16
Pseudarthrobacter sp. NIBRBAC000502771	Af24	17.52	2.02E-12

Af48

Bacterial Taxa	Enriched	Log Fold	P-adjust
Massilia plicata	A1	-21.49	1.45E-10
Metabacillus indicus	A1	-3.36	2.17E-04
Peribacillus simplex	A1	-17.36	5.00E-03
Solibacillus silvestris	A1	-18.34	1.06E-06
Stenotrophomonas sp. G4	A1	-27.73	7.93E-36
Azospirillum sp. TSA2s	A1	-16.36	4.13E-03
Exiguobacterium acetylicum	A1	-23.24	6.13E-14
Exiguobacterium sp. U13-1	A1	-23.57	2.85E-14
Paenibacillus sp. 37	A1	-21.25	3.34E-12
Paenibacillus xylanexedens	A1	-21.19	9.35E-11
Planomicrobium chinense	A1	-21.48	6.42E-09
Flavisolibacter tropicus	Af48	3.44	5.00E-03
Paucimonas lemoignei	Af48	18.26	4.06E-03
Arthrobacter sp. KBS0702	Af48	7.72	3.21E-04
Pseudarthrobacter phenanthrenivorans	Af48	18.26	7.54E-19
Adhaeribacter swui	Af48	17.59	5.30E-19
Pseudarthrobacter sp. NIBRBAC000502771	Af48	18.09	5.80E-15

Enriched column shows which treatment the bacterial taxa is enriched (A1: single alfalfa plant, Af2: single alfalfa and fescue plant, Af24: 12 alfalfa and fescue plants, Af48: 24 alfalfa and fescue plants). Bacterial taxa which were enriched when alfalfa was grown alone as compared to multiple density treatments. Bacterial taxa which were enriched in only one treatment of increasing plant density is highlighted in orange. Bacterial taxa which were enriched in more than one diversity treatment is highlighted in light sky blue. Bacterial taxa which were enriched all density treatment is highlighted in sky blue.
