# Supplementary material for: Effect of intra- and inter-specific plant interactions on the rhizosphere microbiome of a single target plant at different densities
Source: PLoS One. 2025 Jan 27;20(1):e0316676. doi: 10.1371/journal.pone.0316676 (PMC11771940; doi:10.1371/journal.pone.0316676)
Supplement: S5 Table — Enriched column shows which treatment the bacterial taxa is enriched (A1: single alfalfa plant, Abf2: single alfalfa, brassica, and fescue plant, Abf24: 8 alfalfa, brassica, and fescue plants, Af48: 16 alfalfa, brassica, and fescue plants). Bacterial taxa which were enriched when alfalfa was grown alone as compared to multiple density treatments. Bacterial taxa which were enriched in only one treatment of increasing plant density is highlighted in orange. Bacterial taxa which were enriched in more than one diversity treatment is highlighted in light sky blue. Bacterial taxa which were enriched all density treatment is highlighted in sky blue. (PDF) [file pone.0316676.s006.pdf]

**S5 Table. Differential abundance comparison of alfalfa when grown alone (1 plant) and alfalfa-brassica-fescue mixtures.**

**Abf3**

| Bacterial Taxa | Enriched | Log Fold | P-adjust |
|---|---|---|---|
| *Exiguobacterium aurantiacum* | A1 | -19.23 | 7.32E-09 |
| *Larkinella insperata* | A1 | -21.27 | 3.07E-04 |
| *Halomicronema hongdechloris* | A1 | -20.27 | 1.23E-03 |
| *Adhaeribacter aerophilus* | Abf3 | 16.67 | 5.19E-06 |
| *Adhaeribacter swui* | Abf3 | 20.30 | 2.07E-25 |
| *Pseudarthrobacter* sp. NIBRBAC000502771 | Abf3 | 14.59 | 3.29E-09 |
| *Pseudarthrobacter phenanthrenivorans* | Abf3 | 14.37 | 6.88E-11 |

**Abf24**

| Bacterial Taxa | Enriched | Log Fold | P-adjust |
|---|---|---|---|
| *Paenibacillus xylanexedens* | A1 | -19.80 | 4.56E-09 |
| *Planctomyces* sp. SH-PL14 | A1 | -19.66 | 1.30E-05 |
| *Arthrobacter* sp. KBS0702 | Abf24 | 7.59 | 9.20E-04 |
| *Adhaeribacter aerophilus* | Abf24 | 13.02 | 1.77E-03 |
| *Adhaeribacter swui* | Abf24 | 17.74 | 2.78E-19 |
| *Pseudarthrobacter* sp. NIBRBAC000502771 | Abf24 | 19.22 | 6.70E-17 |
| *Pseudarthrobacter phenanthrenivorans* | Abf24 | 18.53 | 2.78E-19 |

**Abf48**

| Bacterial Taxa | Enriched | Log Fold | P-adjust |
|---|---|---|---|
| *Azospirillum brasilense* | A1 | -24.73 | 3.48E-13 |
| *Azospirillum* sp. TSH58 | A1 | -20.80 | 9.12E-06 |
| *Exiguobacterium sibiricum* | A1 | -5.99 | 9.10E-03 |
| *Exiguobacterium undae* | A1 | -6.34 | 3.74E-04 |
| *Leptolyngbya* sp. O-77 | A1 | -19.31 | 4.38E-04 |
| *Methylophilus* sp. TWE2 | A1 | -21.39 | 1.19E-04 |
| *Oscillatoria nigro-viridis* | A1 | -23.79 | 6.89E-14 |
| *Paenibacillus* sp. 37 | A1 | -21.67 | 1.56E-12 |
| *Peribacillus simplex* | A1 | -17.70 | 4.17E-03 |
| *Planomicrobium chinense* | A1 | -21.86 | 5.45E-09 |
| *Roseimicrobium gellanilyticum* | A1 | -20.28 | 2.01E-07 |
| *Trichocoleus desertorum* | A1 | -20.95 | 1.77E-05 |
| *Halomicronema hongdechloris* | A1 | -20.09 | 7.63E-04 |
| *Planctomyces* sp. SH-PL14 | A1 | -21.54 | 5.45E-07 |
| *Arthrobacter* sp. KBS0702 | Abf48 | 8.57 | 3.70E-05 |
| *Adhaeribacter aerophilus* | Abf48 | 13.97 | 2.51E-04 |
| *Adhaeribacter swui* | Abf48 | 16.98 | 1.66E-17 |
| *Pseudarthrobacter* sp. NIBRBAC000502771 | Abf48 | 17.66 | 5.31E-14 |
| *Pseudarthrobacter phenanthrenivorans* | Abf48 | 18.40 | 1.33E-18 |

Enriched column shows which treatment the bacterial taxa is enriched (A1: single alfalfa plant, Abf2: single alfalfa, brassica, and fescue plant, Abf24: 8 alfalfa, brassica, and fescue plants, Af48: 16 alfalfa, brassica, and fescue plants). Bacterial taxa which were enriched when alfalfa was grown alone as compared to multiple density treatments. Bacterial taxa which were enriched in only one treatment of increasing plant density is highlighted in orange. Bacterial taxa which were enriched in more than one diversity treatment is highlighted in light sky blue. Bacterial taxa which were enriched all density treatment is highlighted in sky blue.
