# Supplementary material for: Effect of intra- and inter-specific plant interactions on the rhizosphere microbiome of a single target plant at different densities
Source: PLoS One. 2025 Jan 27;20(1):e0316676. doi: 10.1371/journal.pone.0316676 (PMC11771940; doi:10.1371/journal.pone.0316676)
Supplement: S6 Table — Enriched column shows which treatment the bacterial taxa is enriched (B1: single brassica plant, Ba2: single brassica and alfalfa plants, B24: 24 brassica plants, B48: 48 brassica plants). Bacterial taxa which were enriched when brassica was grown alone as compared to multiple density treatments. Bacterial taxa which were enriched in only one treatment of increasing plant density is highlighted in orange. Bacterial taxa which were enriched in more than one diversity treatment is highlighted in light sky blue. Bacterial taxa which were enriched all density treatment is highlighted in sky blue. (PDF) [file pone.0316676.s007.pdf]

S6 Table. Differential Abundance Comparison of brassica when grown alone (1 plant) and brassica plant densities.

B24

Bacterial Taxa	Enriched	Log Fold	P-adjust
Anabaena cylindrica	B1	-25.48	7.77E-03
Aneurinibacillus soli	B1	-22.03	2.20E-03
Halomicronema hongdechloris	B1	-24.41	1.36E-03
Leptolyngbya boryana	B1	-21.67	7.32E-03
Paenibacillus odorifer	B1	-20.30	3.97E-04
Paenibacillus sp. FSL H7-0357	B1	-19.47	9.55E-03
Nocardioides alpinus	B24	18.50	6.35E-04
Nocardioides cavernae	B24	19.14	7.50E-08
Solibacillus silvestris	B24	19.32	1.42E-04

B48

Bacterial Taxa	Enriched	Log Fold	P-adjust
Pontibacter populi	B1	-7.88	1.65E-04
Tumebacillus flagellatus	B48	7.75	1.24E-07
Nocardioides alpinus	B48	18.21	7.89E-04
Nocardioides cavernae	B48	18.51	1.67E-07
Solibacillus silvestris	B48	19.40	9.28E-05

Enriched column shows which treatment the bacterial taxa is enriched (B1: single brassica plant, Ba2: single brassica and alfalfa plants, B24: 24 brassica plants, B48: 48 brassica plants). Bacterial taxa which were enriched when brassica was grown alone as compared to multiple density treatments. Bacterial taxa which were enriched in only one treatment of increasing plant density is highlighted in orange. Bacterial taxa which were enriched in more than one diversity treatment is highlighted in light sky blue. Bacterial taxa which were enriched all density treatment is highlighted in sky blue.
