# Supplementary material for: Effect of intra- and inter-specific plant interactions on the rhizosphere microbiome of a single target plant at different densities
Source: PLoS One. 2025 Jan 27;20(1):e0316676. doi: 10.1371/journal.pone.0316676 (PMC11771940; doi:10.1371/journal.pone.0316676)
Supplement: S7 Table — Enriched column shows which treatment the bacterial taxa is enriched (B1: single brassica plant, Ba24: 12 brassica and alfalfa plants, Ba48: 24 brassica and alfalfa plants). Bacterial taxa which were enriched when brassica was grown alone as compared to multiple density treatments. Bacterial taxa which were enriched in only one treatment of increasing plant density is highlighted in orange. Bacterial taxa which were enriched in more than one diversity treatment is highlighted in light sky blue. Bacterial taxa which were enriched all density treatment is highlighted in sky blue. (PDF) [file pone.0316676.s008.pdf]

S7 Table. Differential abundance comparison of brassica when grown alone (1 plant) and brassica-alfalfa mixtures.

Ba2

Bacterial Taxa	Enriched	Log Fold	P-adjust
Aneurinibacillus soli	B1	-21.67	2.93E-05
Azospirillum sp. TSH58	B1	-.23.08	1.11E-10
Domibacillus robiginosus	B1	-18.17	1.35E-13
Exiguobacterium aurantiacum	B1	-24.54	9.03E-20
Pseudarthrobacter sp. NIBRBAC000502771	B1	-7.96	1.66E-04
Anabaena cylindrica	B1	-25.21	1.20E-04
Paenibacillus sp. FSL H7-0357	B1	-19.22	1.71E-04
Spirosoma linguale	Ba2	18.90	1.53E-04
Nocardioides alpinus	Ba2	17.23	1.89E-05
Nocardioides cavernae	Ba2	17.69	3.04E-09
Solibacillus silvestris	Ba2	19.39	4.24E-07

Ba24

Bacterial Taxa	Enriched	Log Fold	P-adjust
Adhaeribacter aerophilus	B1	-17.55	9.95E-13
Daejeonella composti	B1	-25.64	1.62E-08
Ensifer adhaerens	B1	-16.90	3.33E-04
Massilia plicata	B1	-20.09	3.13E-12
Methylotenera mobilis	B1	-7.26	3.07E-06
Paenibacillus xylanexedens	B1	-21.99	1.65E-16
Pirellula sp. SH-Sr6A	B1	-9.45	9.17E-05
Pontibacter populi	B1	-7.56	2.69E-06
Pontibacter rhizosphera	B1	-24.29	4.72E-10
Rhizobacter gummiphilus	B1	-22.21	1.86E-04
Roseomonas ludipueritiae	B1	-20.58	2.64E-04
Sinorhizobium fredii	B1	-18.59	6.57E-07
Telluribacter humicola	B1	-20.17	5.52E-06
Trichocoleus desertorum	B1	-25.81	2.57E-13
Halomicronema hongdechloris	B1	-24.22	1.31E-05
Larkinella arboricola	B1	-20.75	3.76E-04
Larkinella insperata	B1	-21.14	9.13E-06
Leptolyngbya boryana	B1	-20.96	1.42E-04
Lysobacter soli	B1	-24.72	3.74E-04
Paenibacillus sp. FSL H7-0357	B1	-18.94	2.12E-04
Metabacillus indicus	Ba24	4.38	1.07E-10
Pseudomonas stutzeri	Ba24	18.65	4.42E-04
Nocardioides alpinus	Ba24	15.74	1.04E-04
Nocardioides cavernae	Ba24	19.99	1.80E-11
Solibacillus silvestris	Ba24	20.04	1.71E-07

Ba48

Bacterial Taxa	Enriched	Log Fold	P-adjust
Exiguobacterium sibiricum	B1	-4.68	4.13E-03
Exiguobacterium sp. MH3	B1	-8.38	3.20E-03
Exiguobacterium undae	B1	-4.97	9.69E-04
Prosthecobacter fluviatilis	B1	-22.62	2.80E-07
Stenotrophomonas sp. G4	B1	-9.69	1.37E-03
Stenotrophomonas sp. MYb57	B1	-23.06	1.08E-13
Anabaena cylindrica	B1	-25.68	3.66E-03
Halomicronema hongdechloris	B1	-24.47	9.25E-04
Larkinella arboricola	B1	-21.22	9.75E-03
Larkinella insperata	B1	-20.92	9.25E-04
Leptolyngbya boryana	B1	-21.94	3.20E-03
Lysobacter soli	B1	-25.10	9.75E-03
Bacillus sp. Y1	Ba48	3.65	4.25E-04
Metabacillus litoralis	Ba48	2.44	9.75E-03
Spirosoma linguale	Ba48	19.90	3.20E-03
Nocardioides alpinus	Ba48	17.36	1.00E-03
Nocardioides cavernae	Ba48	17.33	1.15E-06
Solibacillus silvestris	Ba48	20.24	1.83E-05

Enriched column shows which treatment the bacterial taxa is enriched (B1: single brassica plant, Ba24: 12 brassica and alfalfa plants, Ba48: 24 brassica and alfalfa plants). Bacterial taxa which were enriched when brassica was grown alone as compared to multiple density treatments. Bacterial taxa which were enriched in only one treatment of increasing plant density is highlighted in orange. Bacterial taxa which were enriched in more than one diversity treatment is highlighted in light sky blue. Bacterial taxa which were enriched all density treatment is highlighted in sky blue.
