# Supplementary material for: Effect of intra- and inter-specific plant interactions on the rhizosphere microbiome of a single target plant at different densities
Source: PLoS One. 2025 Jan 27;20(1):e0316676. doi: 10.1371/journal.pone.0316676 (PMC11771940; doi:10.1371/journal.pone.0316676)
Supplement: S8 Table — Enriched column shows which treatment the bacterial taxa is enriched (B1: single brassica plant, Bf1: single brassica and fescue plants, Bf24: 12 brassica and fescue plants, Bf48: 24 brassica and fescue plants). Bacterial taxa which were enriched when brassica was grown alone as compared to multiple density treatments. Bacterial taxa which were enriched in only one treatment of increasing plant density is highlighted in orange. Bacterial taxa which were enriched in more than one diversity treatment is highlighted in light sky blue. Bacterial taxa which were enriched all density treatment is highlighted in sky blue. (PDF) [file pone.0316676.s009.pdf]

S8 Table. Differential abundance comparison of brassica when grown alone (1 plant) and brassica-fescue mixtures.

Bf2

Bacterial Taxa	Enriched	Log Fold	P-adjust
Aneurinibacillus soli	B1	-21.27	3.02E-03
Exiguobacterium aurantiacum	B1	-23.78	6.01E-16
Larkinella insperata	B1	-21.17	7.62E-04
Pontibacter populi	B1	-7.48	3.62E-04
Pontibacter rhizosphera	B1	-24.22	1.37E-07
Prosthecobacter fluviatilis	B1	-21.33	1.05E-06
Spirosoma linguale	Bf2	19.25	6.60E-03
Solibacillus silvestris	Bf2	15.39	4.98E-03
Nocardioides cavernae	Bf2	17.57	6.95E-07

Bf24

Bacterial Taxa	Enriched	Log Fold	P-adjust
Daejeonella composti	B1	-23.22891	1.48E-06
Paenarthrobacter nicotinovorans	B1	-19.0309	2.22E-09
Paenibacillus sp. 37	B1	-22.05469	1.92E-15
Paenibacillus xylanexedens	B1	-22.52285	2.56E-15
Nocardioides alpinus	Bf24	17.17779	5.64E-05
Nocardioides cavernae	Bf24	18.14108	8.26E-09

Bf48

Bacterial Taxa	Enriched	Log Fold	P-adjust
Anabaena cylindrica	B1	-25.74	5.50E-03
Halomicronema hongdechloris	B1	-24.49	8.58E-04
Oscillatoria nigro-viridis	B1	-26.77	4.80E-14
Rhizobacter gummiphilus	B1	-23.10	5.80E-03
Sphingoaurantiacus capsulatus	B1	-22.04	1.25E-17
Sphingoaurantiacus polygranulatus	B1	-22.36	1.95E-16
Prosthecobacter fluviatilis	B1	-22.69	9.91E-08
Nocardioides alpinus	Bf48	18.71	3.05E-04
Solibacillus silvestris	Bf48	16.33	1.56E-03
Nocardioides cavernae	Bf48	18.50	7.24E-08

Enriched column shows which treatment the bacterial taxa is enriched (B1: single brassica plant, Bf1: single brassica and fescue plants, Bf24: 12 brassica and fescue plants, Bf48: 24 brassica and fescue plants). Bacterial taxa which were enriched when brassica was grown alone as compared to multiple density treatments. Bacterial taxa which were enriched in only one treatment of increasing plant density is highlighted in orange. Bacterial taxa which were enriched in more than one diversity treatment is highlighted in light sky blue. Bacterial taxa which were enriched all density treatment is highlighted in sky blue.
