# Supplementary material for: Effect of intra- and inter-specific plant interactions on the rhizosphere microbiome of a single target plant at different densities
Source: PLoS One. 2025 Jan 27;20(1):e0316676. doi: 10.1371/journal.pone.0316676 (PMC11771940; doi:10.1371/journal.pone.0316676)
Supplement: S9 Table — Enriched column shows which treatment the bacterial taxa is enriched (B1: single brassica plant, Baf1: single brassica, alfalfa, and fescue plants, Baf24: 8 brassica, alfalfa, and fescue plants, Baf48: 16 brassica, alfalfa, and fescue plants). Bacterial taxa which were enriched when brassica was grown alone as compared to multiple density treatments. Bacterial taxa which were enriched in only one treatment of increasing plant density is highlighted in orange. Bacterial taxa which were enriched in more than one diversity treatment is highlighted in light sky blue. Bacterial taxa which were enriched all density treatment is highlighted in sky blue. (PDF) [file pone.0316676.s010.pdf]

S9 Table. Differential abundance comparison of brassica when grown alone (1 plant) and brassica-alfalfa-fescue mixtures.

Baf3

Bacterial Taxa	Enriched	Log Fold	P-adjust
Daejeonella oryzae	B1	-23.20	1.23E-13
Exiguobacterium aurantiacum	B1	-20.41	1.03E-10
Gemmata massiliana	B1	-21.30	7.67E-09
Gemmata sp. SH-PL17	B1	-21.26	1.61E-05
Larkinella insperata	B1	-23.77	1.71E-05
Prosthecobacter fluviatilis	B1	-19.85	4.00E-06
Sphingoaurantiacus polygranulatus	B1	-20.69	2.49E-12
Daejeonella composti	B1	-20.21	1.25E-04
Pontibacter rhizosphera	B1	-19.68	1.42E-05
Nocardioides alpinus	Baf3	18.59	5.58E-05

Baf24

Bacterial Taxa	Enriched	Log Fold	P-adjust
Aneurinibacillus soli	B1	-20.95	4.56E-03
Daejeonella composti	B1	-25.20	7.92E-06
Pontibacter rhizosphera	B1	-24.00	4.18E-07
Tumebacillus flagellatus	Baf24	4.89	4.56E-03
Nocardioides cavernae	Baf24	16.46	8.50E-06
Solibacillus silvestris	Baf24	19.05	9.46E-05
Nocardioides alpinus	Baf24	18.27	6.21E-04

Baf48

Bacterial Taxa	Enriched	Log Fold	P-adjust
Azospirillum sp. TSH58	B1	-23.14	7.89E-09
Exiguobacterium acetylicum	B1	-22.59	5.47E-20
Exiguobacterium antarcticum	B1	-4.71	3.69E-03
Exiguobacterium sibiricum	B1	-4.44	7.68E-03
Exiguobacterium sp. MH3	B1	-7.89	6.12E-03
Exiguobacterium sp. U13-1	B1	-22.94	2.51E-19
Exiguobacterium undae	B1	-5.19	2.71E-04
Leptolyngbya boryana	B1	-21.33	4.00E-03
Paenibacillus odorifer	B1	-20.06	1.43E-04
Paenibacillus sp. 37	B1	-22.36	1.43E-15
Paenibacillus sp. FSL H7-0357	B1	-19.24	5.81E-03
Paenibacillus xylanexedens	B1	-22.42	5.93E-15
Pseudomonas koreensis	B1	-20.06	8.10E-03
Stenotrophomonas sp. MYb57	B1	-22.33	1.77E-13
Trichocoleus desertorum	B1	-26.03	1.49E-11
Spirosoma linguale	Baf48	19.37	4.00E-03
Nocardioides cavernae	Baf48	18.64	2.64E-08
Solibacillus silvestris	Baf48	18.12	1.43E-04
Nocardioides alpinus	Baf48	18.24	2.71E-04

Enriched column shows which treatment the bacterial taxa is enriched (B1: single brassica plant, Baf1: single brassica, alfalfa, and fescue plants, Baf24: 8 brassica, alfalfa, and fescue plants, Baf48: 16 brassica, alfalfa, and fescue plants). Bacterial taxa which were enriched when brassica was grown alone as compared to multiple density treatments. Bacterial taxa which were enriched in only one treatment of increasing plant density is highlighted in orange. Bacterial taxa which were enriched in more than one diversity treatment is highlighted in light sky blue. Bacterial taxa which were enriched all density treatment is highlighted in sky blue.
