# Supplementary material for: Effect of intra- and inter-specific plant interactions on the rhizosphere microbiome of a single target plant at different densities
Source: PLoS One. 2025 Jan 27;20(1):e0316676. doi: 10.1371/journal.pone.0316676 (PMC11771940; doi:10.1371/journal.pone.0316676)
Supplement: S10 Table — Enriched column shows which treatment the bacterial taxa is enriched (F1: single fescue plant, F24: 24 fescue plants, F48: 48 fescue plants). Bacterial taxa which were enriched when fescue was grown alone as compared to multiple density treatments. Bacterial taxa which were enriched in only one treatment of increasing plant density is highlighted in orange. Bacterial taxa which were enriched in more than one diversity treatment is highlighted in light sky blue. Bacterial taxa which were enriched all density treatment is highlighted in sky blue. (PDF) [file pone.0316676.s011.pdf]

S10 Table. Differential abundance comparison of fescue when grown alone (1 plant) and fescue plant densities.

F24				F48			
Bacterial Taxa	Enriched	Log Fold	P-adjust	Bacterial Taxa	Enriched	Log Fold	P-adjust
Telluribacter humicola	F1	-19.97	5.94E-06	*Kaistia defluvii*	F1	-24.00	2.26E-08
Lysobacter helvus	F1	-22.62	7.67E-04	*Pseudomonas stutzeri*	F1	-22.96	2.03E-08
Lysobacter sp. TY2-98	F1	-21.69	2.12E-03	*Adhaeribacter terreus*	F48	8.53	7.45E-04
Metabacillus indicus	F24	2.64	7.35E-03	*Adhaeribacter swui*	F48	5.51	6.84E-03
Sinorhizobium meliloti	F24	7.52	7.08E-03	*Dyadobacter sediminis*	F48	18.80	1.23E-07
Trichormus azollae	F24	20.06	8.95E-03	*Ensifer adhaerens*	F48	16.76	7.45E-04
Adhaeribacter aerophilus	F24	14.77	5.94E-06	*Adhaeribacter aerophilus*	F48	15.30	1.25E-06
[Brevibacterium] frigoritolerans	F24	18.96	7.67E-04	*[Brevibacterium] frigoritolerans*	F48	20.12	1.60E-04
Larkinella arboricola	F24	19.42	3.03E-05	*Larkinella arboricola*	F48	18.29	8.47E-05
Larkinella insperata	F24	19.98	7.80E-07	*Larkinella insperata*	F48	18.63	2.32E-06
Paenibacillus sp. 37	F24	17.39	5.86E-10	*Paenibacillus* sp. 37	F48	17.15	9.81E-10

Enriched column shows which treatment the bacterial taxa is enriched (F1: single fescue plant, F24: 24 fescue plants, F48: 48 fescue plants). Bacterial taxa which were enriched when fescue was grown alone as compared to multiple density treatments. Bacterial taxa which were enriched in only one treatment of increasing plant density is highlighted in orange. Bacterial taxa which were enriched in more than one diversity treatment is highlighted in light sky blue. Bacterial taxa which were enriched all density treatment is highlighted in sky blue.
