# Supplementary material for: Effect of intra- and inter-specific plant interactions on the rhizosphere microbiome of a single target plant at different densities
Source: PLoS One. 2025 Jan 27;20(1):e0316676. doi: 10.1371/journal.pone.0316676 (PMC11771940; doi:10.1371/journal.pone.0316676)
Supplement: S11 Table — Enriched column shows which treatment the bacterial taxa is enriched (F1: single fescue plant, Fa1: single fescue and alfalfa plants, Fa24: 12 fescue and alfalfa plants, Fa48: 24 fescue and alfalfa plants). Bacterial taxa which were enriched when fescue was grown alone as compared to multiple density treatments. Bacterial taxa which were enriched in only one treatment of increasing plant density is highlighted in orange. Bacterial taxa which were enriched in more than one diversity treatment is highlighted in light sky blue. Bacterial taxa which were enriched all density treatment is highlighted in sky blue. (PDF) [file pone.0316676.s012.pdf]

S11 Table. Differential abundance comparison of fescue when grown alone (1 plant) and fescue-alfalfa mixtures.

Fa2

Bacterial Taxa	Enriched	Log Fold	P-adjust
Anabaena cylindrica	F1	-23.61	1.94E-09
Azospirillum brasilense	F1	-20.75	3.79E-08
Azospirillum sp. TSH58	F1	-20.48	3.72E-05
Calothrix sp. PCC 7507	F1	-17.47	2.44E-03
Halomicronema hongdechloris	F1	-18.51	5.70E-06
Peribacillus muralis	F1	-7.54	2.72E-03
Peribacillus simplex	F1	-17.07	9.67E-04
Sinorhizobium meliloti	Fa2	7.81	3.72E-03
Ensifer adhaerens	Fa2	16.80	1.24E-03
Larkinella arboricola	Fa2	17.97	2.08E-04
Larkinella insperata	Fa2	17.38	3.72E-05

Fa24

Bacterial Taxa	Enriched	Log Fold	P-adjust
Lysobacter helvus	F1	-21.88	7.45E-04
Lysobacter sp. TY2-98	F1	-20.78	2.58E-03
Noviherbaspirillum malthae	F1	-7.31	7.37E-03
Pararcticibacter amylolyticus	F1	-18.75	1.40E-05
Phenylobacterium haematophilum	F1	-8.10	9.63E-05
Pseudomonas stutzeri	F1	-21.91	7.67E-08
Pseudoxanthomonas mexicana	F1	-22.30	1.44E-05
Telluribacter humicola	F1	-18.64	2.04E-05
Trichormus azollae	Fa24	20.99	3.35E-03
[Brevibacterium] frigoritolerans	Fa24	15.99	6.63E-03
Paenibacillus sp. 37	Fa24	20.29	4.50E-14
Dyadobacter sediminis	Fa24	19.97	2.48E-08
Adhaeribacter aerophilus	Fa24	16.71	7.67E-08
Ensifer adhaerens	Fa24	19.30	4.07E-05
Larkinella arboricola	Fa24	18.57	4.60E-05
Larkinella insperata	Fa24	18.80	2.39E-06

Fa48

Bacterial Taxa	Enriched	Log Fold	P-adjust
Azospirillum sp. TSA2s	F1	-21.84	2.32E-05
Nostoc flagelliforme	F1	-21.72	6.09E-03
Stenotrophomonas sp. G4	F1	-22.37	6.45E-29
Sinorhizobium meliloti	Fa48	8.19	1.54E-03
Adhaeribacter aerophilus	Fa48	15.73	5.86E-07
Dyadobacter sediminis	Fa48	17.57	1.12E-06
Paenibacillus sp. 37	Fa48	16.81	1.47E-09
Ensifer adhaerens	Fa48	14.87	6.09E-03
Larkinella arboricola	Fa48	19.06	2.85E-05
Larkinella insperata	Fa48	19.82	5.58E-07

Enriched column shows which treatment the bacterial taxa is enriched (F1: single fescue plant, Fa1: single fescue and alfalfa plants, Fa24: 12 fescue and alfalfa plants, Fa48: 24 fescue and alfalfa plants). Bacterial taxa which were enriched when fescue was grown alone as compared to multiple density treatments. Bacterial taxa which were enriched in only one treatment of increasing plant density is highlighted in orange. Bacterial taxa which were enriched in more than one diversity treatment is highlighted in light sky blue. Bacterial taxa which were enriched all density treatment is highlighted in sky blue.
