# Supplementary material for: Effect of intra- and inter-specific plant interactions on the rhizosphere microbiome of a single target plant at different densities
Source: PLoS One. 2025 Jan 27;20(1):e0316676. doi: 10.1371/journal.pone.0316676 (PMC11771940; doi:10.1371/journal.pone.0316676)
Supplement: S12 Table — Enriched column shows which treatment the bacterial taxa is enriched (F1: single fescue plant, Fb1: single fescue and brassica plants, Fb24: 12 fescue and brassica plants, Fb48: 24 fescue and brassica plants). Bacterial taxa which were enriched when fescue was grown alone as compared to multiple density treatments. Bacterial taxa which were enriched in only one treatment of increasing plant density is highlighted in orange. Bacterial taxa which were enriched in more than one diversity treatment is highlighted in light sky blue. Bacterial taxa which were enriched all density treatment is highlighted in sky blue. (PDF) [file pone.0316676.s013.pdf]

S12 Table. Differential abundance comparison of fescue when grown alone (1 plant) and fescue-brassica mixtures.

Fb2

Bacterial Taxa	Enriched	Log Fold	P-adjust
Lysobacter helvus	F1	-21.99	1.23E-03
Lysobacter sp. TY2-98	F1	-20.69	4.37E-03
Azohydromonas australica	F1	-22.04	1.97E-03
Calothrix sp. PCC 7507	F1	-18.91	6.96E-04
Ensifer adhaerens	Fb2	20.85	1.35E-05
Adhaeribacter aerophilus	Fb2	15.95	8.42E-07
Dyadobacter sediminis	Fb2	18.17	8.42E-07
Larkinella arboricola	Fb2	17.29	5.05E-04
Larkinella insperata	Fb2	17.78	1.63E-05
Paenibacillus sp. 37	Fb2	20.05	1.26E-13

Fb24

Bacterial Taxa	Enriched	Log Fold	P-adjust
Anabaena cylindrica	F1	-24.97	2.26E-09
Azospirillum sp. TSA2s	F1	-22.24	1.01E-04
Azospirillum sp. TSH58	F1	-21.42	5.94E-05
Calothrix sp. PCC 7507	F1	-18.38	2.42E-03
[Brevibacterium] frigoritolerans	Fb24	20.90	3.08E-04
Adhaeribacter aerophilus	Fb24	15.25	1.38E-05
Dyadobacter sediminis	Fb24	14.96	4.12E-04
Larkinella arboricola	Fb24	15.13	8.77E-03
Larkinella insperata	Fb24	16.49	3.08E-04
Paenibacillus sp. 37	Fb24	16.27	8.37E-08

Fb48

Bacterial Taxa	Enriched	Log Fold	P-adjust
Anabaena cylindrica	F1	-24.75	3.70E-11
Leptolyngbya sp. O-77	F1	-23.95	2.27E-11
Nostoc flagelliforme	F1	-22.85	1.96E-03
Oscillatoria nigro-viridis	F1	-26.59	7.75E-23
Calothrix sp. PCC 7507	F1	-20.36	6.17E-05
Azohydromonas australica	F1	-22.99	4.75E-04
[Brevibacterium] frigoritolerans	Fb48	15.83	6.88E-03
Ensifer adhaerens	Fb48	19.07	4.32E-05
Adhaeribacter aerophilus	Fb48	15.45	5.67E-07
Dyadobacter sediminis	Fb48	18.89	7.28E-08
Larkinella arboricola	Fb48	20.09	5.48E-06
Larkinella insperata	Fb48	20.81	6.40E-08
Paenibacillus sp. 37	Fb48	14.65	2.32E-07

Enriched column shows which treatment the bacterial taxa is enriched (F1: single fescue plant, Fb1: single fescue and brassica plants, Fb24: 12 fescue and brassica plants, Fb48: 24 fescue and brassica plants). Bacterial taxa which were enriched when fescue was grown alone as compared to multiple density treatments. Bacterial taxa which were enriched in only one treatment of increasing plant density is highlighted in orange. Bacterial taxa which were enriched in more than one diversity treatment is highlighted in light sky blue. Bacterial taxa which were enriched all density treatment is highlighted in sky blue.
