# Supplementary material for: Effect of intra- and inter-specific plant interactions on the rhizosphere microbiome of a single target plant at different densities
Source: PLoS One. 2025 Jan 27;20(1):e0316676. doi: 10.1371/journal.pone.0316676 (PMC11771940; doi:10.1371/journal.pone.0316676)
Supplement: S13 Table — Enriched column shows which treatment the bacterial taxa is enriched (F1: single fescue plant, Fab1: single fescue, alfalfa, and brassica plants, Fab24: 8 fescue, alfalfa, and brassica plants, Fab48: 16 fescue, alfalfa, and brassica plants). Bacterial taxa which were enriched when fescue was grown alone as compared to multiple density treatments. Bacterial taxa which were enriched in only one treatment of increasing plant density is highlighted in orange. Bacterial taxa which were enriched in more than one diversity treatment is highlighted in light sky blue. Bacterial taxa which were enriched all density treatment is highlighted in sky blue. (PDF) [file pone.0316676.s014.pdf]

S13 Table. Differential abundance comparison of fescue when grown alone (1 plant) and fescue-alfalfa-brassica mixtures.

Fba3

Bacterial Taxa	Enriched	Log Fold	P-adjust
Planctomyces sp. SH-PL14	F1	-24.16	2.55E-10
Planomicrobium chinense	F1	-20.01	8.77E-09
Calothrix sp. PCC 7507	F1	-19.38	7.11E-04
Nostoc flagelliforme	F1	-22.24	9.13E-03
Adhaeribacter terreus	Fab3	8.42	2.50E-03
Adhaeribacter swui	Fab3	5.80	8.52E-03
Paenibacillus sp. 37	Fab3	19.72	5.57E-13
Adhaeribacter aerophilus	Fab3	18.42	2.54E-09
Dyadobacter sediminis	Fab3	17.29	3.99E-06

Fab24

Bacterial Taxa	Enriched	Log Fold	P-adjust
Daejeonella oryzae	F1	-19.11	5.81E-06
Leptolyngbya sp. O-77	F1	-23.64	1.29E-10
[Brevibacterium] frigoritolerans	Fab24	17.49	2.80E-03
Trichormus azollae	Fab24	20.50	7.39E-03
Paenibacillus sp. 37	Fab24	17.33	3.63E-10
Ensifer adhaerens	Fab24	19.02	7.16E-05
Larkinella arboricola	Fab24	18.96	4.23E-05
Larkinella insperata	Fab24	20.29	2.31E-07
Adhaeribacter aerophilus	Fab24	14.58	5.97E-06
Dyadobacter sediminis	Fab24	19.49	4.89E-08

Fab48

Bacterial Taxa	Enriched	Log Fold	P-adjust
Azospirillum brasilense	F1	-22.10	1.13E-09
Limisphaera ngatamarikiensis	F1	-3.43	3.00E-04
Oscillatoria nigro-viridis	F1	-25.25	2.55E-20
Calothrix sp. PCC 7507	F1	-19.37	2.68E-04
Nostoc flagelliforme	F1	-22.17	4.23E-03
Leptolyngbya sp. O-77	F1	-23.55	8.80E-11
Planomicrobium chinense	F1	-19.94	5.62E-09
Planctomyces sp. SH-PL14	F1	-24.22	1.02E-10
Sinorhizobium meliloti	Fab48	8.81	3.23E-04
Ensifer adhaerens	Fab48	19.73	2.81E-05
Larkinella arboricola	Fab48	17.71	1.74E-04
Larkinella insperata	Fab48	18.35	3.84E-06
Adhaeribacter aerophilus	Fab48	15.84	4.34E-07
Dyadobacter sediminis	Fab48	17.20	2.27E-06

Enriched column shows which treatment the bacterial taxa is enriched (F1: single fescue plant, Fab1: single fescue, alfalfa, and brassica plants, Fab24: 8 fescue, alfalfa, and brassica plants, Fab48: 16 fescue, alfalfa, and brassica plants). Bacterial taxa which were enriched when fescue was grown alone as compared to multiple density treatments. Bacterial taxa which were enriched in only one treatment of increasing plant density is highlighted in orange. Bacterial taxa which were enriched in more than one diversity treatment is highlighted in light sky blue. Bacterial taxa which were enriched all density treatment is highlighted in sky blue.
