# Supplementary material for: Effect of intra- and inter-specific plant interactions on the rhizosphere microbiome of a single target plant at different densities
Source: PLoS One. 2025 Jan 27;20(1):e0316676. doi: 10.1371/journal.pone.0316676 (PMC11771940; doi:10.1371/journal.pone.0316676)
Supplement: S14 Table — (PDF) [file pone.0316676.s015.pdf]

S14 Table. Bacterial taxa characterization for alfalfa and brassica related microbes.

Bacterial Taxa	Characterization	Citation
Adhaeribacter swui	Tested positive for oxidase and catalase	Kim et al. [60]
Neorhizobium sp. SOG26	Metal ion binding properties	NCBI 2060726 uniprot.org
Pseudarthrobacter sp. NIBRBAC000502771	Capable of producing auxin (Indol-3-glycerol phosphate synthase) and has heavy metal (copper and arsenic) resistance	Park et al. [61]
Pseudarthrobacter phenanthrenivornans	Produces several phytohormones (abscisic acid, auxin, cytokinin, ethylene, gibberellins, jasmonic acid, and salicylic acid)	Tshishonga et al. [62]
Pseudarthrobacter oxydans	Synthesizes indole-3-acetic acid, fix nitrogen (ammonia production), solubilize phosphorus, and is resistant to heavy metals (cadmium, copper, and nickel)	Bushra et al. [63]
Paucimonas lemoignei	Flagellated and a potential nitrogen fixer	Jendrossek et al. [64]
Arthrobacter sp. UKPF54-2	Promotes growth for *Brassica* sp., fix nitrogen, produce acetolactate synthase (dihydroxy acid dehydratase and ketol acid reductoisomerase), and produce five genes attributed to antimicrobial properties	Shen et al. [65]
Adhaeribacter aerophilus	Oxidase positive and may hydrolyze starch	Weon et al. [71]
Larkinella arboricola	Indole producer	Kulichevskaya et al. [72]
Larkinella insperata	Bared no remarkable plant related characteristics	Anandham et al. [73] Vancanneyt et al. [74]
Dyadobacter sediminis	Produces catalase, oxidase, alkaline phosphatase, and acid phosphatase	Tian et al. [75]
Paenibacillus sp. 37	Possible plant growth promotor due to its ability for siderophore biosynthesis, antimicrobial production (paeninodin, bacitracin, paenilipoheptin, xenocoumacin, pellasoren, and octapeptin), and its phytohormone-associated genes	Garcia-Lemos et al. [76]
Brevibacterium frigrotolerans	Shown to solubilize phosphate, produce indole-3-acetic acid, and produce siderophores	Tara et al. [77]
Trichormus azollae	Nitrogen fixer for ferns	Gunawardana et al. [78]
Ensifer adhaerens	Nitrogen fixer and may produce indole-3-acetic acid, exopolysaccharides, ammonia, siderophores, salicylic acid (for abiotic stress), and even promote seed germination for soybean	Zhou et al. [79]
Sinorhizobium meliloti	Nitrogen fixing symbiont of alfalfa	Galibert et al. [80]
