# Supplementary material for: Effect of intra- and inter-specific plant interactions on the rhizosphere microbiome of a single target plant at different densities
Source: PLoS One. 2025 Jan 27;20(1):e0316676. doi: 10.1371/journal.pone.0316676 (PMC11771940; doi:10.1371/journal.pone.0316676)
Supplement: S15 Table — (PDF) [file pone.0316676.s016.pdf]

S15 Table. 1st, 2nd, and 3rd largest module by plant network.

Network	Module 1: 17.31%	Module 2: 17.31%	Module 3: 13.46%
A	*Alkalihalobacillus halodurans*<br>*Bacillus infantis*<br>*Bacillus methanolicus*<br>*Bacillus* sp. S3<br>*Bacillus* sp. X1(2014)<br>*Bacillus* sp. 1NLA3E<br>*Mesobacillus subterraneus*<br>*Mesobacillus foraminis*<br>*Paenibacillus beijingensis*	*Bacillus circulans*<br>*Cohnella abietis*<br>*Cohnella phaseoli*<br>*Cohnella massiliensis*<br>*Cytobacillus oceanisediminis*<br>*Limisphaera ngatamarikiensis*<br>*Paenibacillus swuensis*<br>*Paenibacillus chitinolvticus*<br>*Paenibacillus tyrfis*	*Arthrobacter* sp. KBS0702<br>*Arthrobacter* sp. PGP41<br>*Arthrobacter* sp. QXT-31<br>*Arthrobacter* sp. UKPF54-2<br>*Pseudarthrobacter* sp. NIBRBAC000502771<br>*Pseudarthrobacter phenanthrenivorans*
Network	Module 1: 13.43%	Module 2: 8.96%	Module 3: 8.96%
Ab	*Flavisolibacter galbus*<br>*Flavisolibacter ginsengisoli*<br>*Flavisolibacter ginsenosidimutans*<br>*Microvirga aerilata*<br>*Microvirga ossetica*<br>*Microvirga soli*<br>*Microvirga subterranean*<br>*Microvirga zambiensis*<br>*Microvirga* sp. 17 mud 1-3	*Bacillus* sp. X1(2014)<br>*Bacillus* sp. 1NLA3E<br>*Bacillus* sp. S3<br>*Bacillus infantis*<br>*Bacillus* sp. Y1.<br>*Neobacillus niacin*	*Anabaena cylindrica*<br>*Exiguobacterium antarcticum*<br>*Exiguobacterium sibiricum*<br>*Exiguobacterium undae*<br>*Stenotrophomonas* sp. G4<br>*Stenotrophomonas* sp. MYb57
Network	Module 1: 16.22%	Module 2: 13.51%	Module 3: 8.11%
Af	*Bacillus infantis*<br>*Bacillus mediterraneensis*<br>*Bacillus methanolicus*	*Achromobacter xylosoxidans*<br>*Ensifer adhaerens*	*Massilia agri*<br>*Massilia oculi*<br>*Massilia* sp. WG5

	Bacillus sp. 1NLA3E<br>*Mesobacillus foraminis*<br>*Mesobacillus subterraneus*	*Exiguobacterium mexicanum*<br>*Stenotrophomonas* sp. G4<br>*Stenotrophomonas* sp. MYb57	
Network	Module 1: 14.55%	Module 2: 12.73%	Module 3: 10.91%
Abf	*Massilia agri*<br>*Massilia albidiflava*<br>*Massilia oculi*<br>*Massilia putida*<br>*Massilia timonae*<br>*Massilia umbonata*<br>*Massilia violaceinigra*<br>*Massilia* sp. WG5	*Bacillus acidicola*<br>*Bacillus carboniphilus*<br>*Bacillus mediterraneensis*<br>*Bacillus licheniformis*<br>*Bacillus paralicheniformis*<br>*Mesobacillus foraminis*<br>*Mesobacillus subterraneus*	*Alkalihalobacillus halodurans*<br>*Bacillus circulans*<br>*Bacillus dafuensis*<br>*Bacillus infantis*<br>*Bacillus methanolicus*<br>*Cytobacillus gottheili*
Network	Module 1: 22.41%	Module 2: 13.79%	Module 3: 4.9%
B	*Ammoniphilus resinae*<br>*Alkalihalobacillus halodurans*<br>*Bacillus mediterraneensis*<br>*Bacillus methanolicus*<br>*Bacillus infantis*<br>*Bacillus* sp. 1NLA3E<br>*Cytobacillus gottheilii*<br>*Mesobacillus foraminis*<br>*Mesobacillus subterraneus*<br>*Oxalophagus oxalicus*<br>*Paenibacillus beijingensis*<br>*Paenibacillus chitinolyticus*<br>*Paenibacillus vunnanensis*	*Cohnella abietis*<br>*Cohnella candidum*<br>*Cohnella massiliensis*<br>*Cohnella phaseoli*<br>*Cytobacillus oceanisediminis*<br>*Tumebacillus algifaecis*<br>*Tumebacillus ginsengisoli*<br>*Tumebacillus soli*	4 way tie<br>*Exiguobactrium* sp.<br>*Flavisolibacter, Bacillus* sp.<br>*Microvirga* sp.

Network	Module 1: 14.89%	Module 2: 12.77%	Module 3: 8.51%
Ba	Alkalihalobacillus halodurans<br>Bacillus dafuensis<br>Bacillus mediterraneensis<br>Bacillus methanolicus<br>Cytobacillus gottheilli<br>Paenibacillus beijingensis<br>Paenibacillus chitinolyticus	*Bacillus infantis*<br>*Bacillus* sp. 1NLA3E<br>*Bacillus* sp. Y1.<br>*Cytobacillus Oceanisediminis*<br>*Mesobacillus foraminis*<br>*Mesobacillus litoralis*	*Bacillus* sp. S3<br>*Bacillus* sp. X1(2014)<br>*Neobacillus mesonae*<br>*Neobacillus niacin*
Network	Module 1: 23.33%	Module 2: 18.33%	Module 3: 11.67%
Bf	*Bacillus infantis*<br>*Bacillus mediterraneensis*<br>*Bacillus methanolicus*<br>*Bacillus* sp. S3<br>*Bacillus* sp. X1(2014)<br>*Bacillus* sp. Y1.<br>*Bacillus* sp. 1NLA3E<br>*Cytobacillus gottheilii*<br>*Cytobacillus Oceanisediminis*<br>*Mesobacillus foraminis*<br>*Metabacillus litoralis*<br>*Mesobacillus subterraneus*<br>*Neobacillus niacin*<br>*Neobacillus mesonae*	*Massilia armeniaca*<br>*Massilia namucuonensis*<br>*Massilia putida*<br>*Massilia oculi*<br>*Massilia umbonate*<br>*Massilia violaceinigra*<br>*Microvirga ossetica*<br>*Microvirga subterranean*<br>*Microvirga* sp. 17 mud 1-3<br>*Paracoccus* sp. Arc7-R13	*Exiguobacterium acetylicum*<br>*Exiguobacterium antarcticum*<br>*Exiguobacterium sibiricum*<br>*Exiguobacterium undae*<br>*Exiguobacterium* sp. U13-1<br>*Exiguobacterium* sp. ZWU0009<br>*Flavisolibacter* sp. 17J28-1
Network	Module 1: 18.87%	Module 2: 13.21%	Module 3: 7.55%
Baf	*Alkalihalobacillus halodurans*<br>*Bacillus mediterraneensis*<br>*Bacillus methanolicus*	*Massilia agri*<br>*Massilia alkalitolerans*<br>*Massilia oculi*	*Bacillus* sp. S3<br>*Bacillus* sp. X1(2014)<br>*Bacillus* sp. 1NLA3E

	Devosia sp. A16<br>*Flaviaesturaribacter luteus*<br>*Mesobacillus foraminis*<br>*Mesobacillus stamsii*<br>*Mesobacillus subterraneus*<br>*Paenibacillus chitinolyticus*<br>*Pontibacter populi*	*Massilia putida*<br>*Massilia Umbonata*<br>*Massilia violaceinigra*<br>*Massilia* sp. WG5	*Neobacillus mesonae*
Network	Module 1: 12.5%	Module 2: 9.38%	Module 3: 9.38%
F	*Microvirga ossetica*<br>*Microvirga soli*<br>*Microvirga subterranean*<br>*Microvirga* sp. 17 mud 1-3	*Exiguobacterium antarcticum*<br>*Exiguobacterium sibiricum*<br>*Exiguobacterium undae*	*Noviherbaspirillum aurantiacum*<br>*Noviherbaspirillum massiliense*<br>*Noviherbaspirillum soli*
Network	Module 1: 19.15%	Module 2: 10.64%	Module 3: 8.51%
Fa	*Alkalihalobacillus halodurans*<br>*Bacillus ciccensis*<br>*Bacillus infantis*<br>*Bacillus mediterraneensis*<br>*Bacillus methanolicus*<br>*Cytobacillus gottheili*<br>*Mesobacillus foraminis*<br>*Mesobacillus subterraneus*<br>*Paenibacillus chitinolvticus*	*Massilia agri*<br>*Massilia oculi*<br>*Massilia putida*<br>*Massilia umbonate*<br>*Massilia* sp. WG5	*Herbaspirillum* sp. meg3<br>*Herbaspirillum seropedica*<br>*Noviherbaspirillum aurantiacum*<br>*Noviherbaspirillum soli*
Network	Module 1: 20.73%	Module 2: 14.63%	Module 3: 14.63%
Fb	*Ammoniphilus resinae*<br>*Bacillus infantis*<br>*Bacillus mediterraneensis*	*Massilia albidiflava*<br>*Massilia armeniaca*<br>*Massilia namucuonensis*	*Achromobacter insolitus*<br>*Achromobacter spanius*

	Bacillus methanolicus<br>*Bacillus* sp. S3<br>*Bacillus* sp. X1(2014)<br>*Bacillus* sp. 1NLA3E<br>*Cytobacillus gottheili*<br>*Cytobacillus oceanisediminis*<br>*Mesobacillus foraminis*<br>*Mesobacillus stamsii*<br>*Mesobacillus subterraneus*<br>*Oxalophagus oxalicus*<br>*Paenibacillus mucilaginosus*<br>*Pedobacter mongoliensis*<br>*Roseisolibacter agri*<br>*Rufibacter* sp. DG31D	*Massilia oculi*<br>*Massilia putida*<br>*Massilia umbonate*<br>*Massilia*<br>*Microvirga ossetica*<br>*Microvirga subterrânea*<br>*Microvirga zambiensis*<br>*Microvirga* sp. 17 mud 1-3<br>*Ramlibacter tataouinensis*	*Achromobacter xylosoxidans*<br>*Chthoniobacter flavus*<br>*Flavisolibacter ginsenosidimutans*<br>*Flavisolibacter tropicus*<br>*Metabacillus litoralis*<br>*Methylotenera versatilis*<br>*Pontibacter chitinilvticus*<br>*Pontibacter korlensis*<br>*Pontibacter Populi*<br>*Rubellimicrobium roseum*<br>Fescue-alfalfa-brassica plant mixture
Network	Module 1: 20%	Module 2: 16.67%	Module 3: 10%
Fab	*Bacillus methanolicus*<br>*Bacillus* sp. S3<br>*Bacillus* sp. X1(2014)<br>*Bacillus* sp. 1NLA3E<br>*Neobacillus mesonae*<br>*Paenibacillus yunnanensis*	*Microvirga ossetica*<br>*Microvirga soli*<br>*Microvirga subterranean*<br>*Microvirga zambiensis*<br>*Rubellimicrobium roseum*	5 way tie
