# Supplementary material for: Effect of intra- and inter-specific plant interactions on the rhizosphere microbiome of a single target plant at different densities
Source: PLoS One. 2025 Jan 27;20(1):e0316676. doi: 10.1371/journal.pone.0316676 (PMC11771940; doi:10.1371/journal.pone.0316676)
Supplement: S16 Table — First capitalized letter denotes plant species rhizosphere; lower case letter denotes neighboring plant species. (PDF) [file pone.0316676.s017.pdf]

S16 Table. Bacteriome Network Statistics.

Network	Node	Edge	Avg. Weight Degree	Graph Density	Connected Components	Modularity	Avg. Clustering Coefficient	Avg. Path Length
A	52	58	2.037	0.044	12	0.858	0.627	2.065
Ab	71	56	1.577	0.023	27	0.898	0.576	1.71
Af	42	26	1.141	0.03	19	0.876	0.515	1.537
Abf	55	49	1.597	0.033	18	0.873	0.636	1.54
B	58	80	2.454	0.048	13	0.714	0.678	2.88
Ba	47	38	1.454	0.035	15	0.861	0.525	2.184
Bf	60	99	2.993	0.056	10	0.761	0.688	2.942
Baf	53	54	1.822	0.039	15	0.819	0.676	2.139
F	32	21	1.206	0.042	14	0.898	0.926	1.087
Fa	47	35	1.34	0.032	16	0.874	0.473	1.775
Fb	82	136	2.948	0.041	10	0.682	0.478	5.331
Fab	30	26	1.556	0.06	9	0.835	0.562	1.452

First capitalized letter denotes plant species rhizosphere; lower case letter denotes neighboring plant species.
