# Supplementary material for: Effect of intra- and inter-specific plant interactions on the rhizosphere microbiome of a single target plant at different densities
Source: PLoS One. 2025 Jan 27;20(1):e0316676. doi: 10.1371/journal.pone.0316676 (PMC11771940; doi:10.1371/journal.pone.0316676)
Supplement: Attachment — Submitted filename: Response_PLOSOne.docx [file pone.0316676.s018.docx]

**We thank the editors and the reviews for their feedback. The major concern was with the similarity of another study that has been published. We have added the results obtained in that previous manuscript in the current manuscript. In the introduction and the materials and methods we have clarified that they are the different samples from the same experiment as Sci Rep 13, 17150 (2023). https://doi.org/10.1038/s41598-023-44104-8.**

The first manuscript looked at plant biomass and the bacteriome of the **bulk** soil to investigate the impact of increasing plant density and diversity can have on the soil prior to the next cropping cycle. It was found that even in high plant competition, overall forage biomass can increase, and that different plant mixtures produced different bulk soil bacteriomes. However, increasing plant diversity and density hardly guarantees and increase in bacterial diversity or abundance. In all, it shows that agriculturalists may increase plant biomass that will be incorporated in the soil for the next cropping cycle, and adding different plant species will influence what bacteria are present in the bulk soil.

**The current study** addresses a basic science question. It is known that plants recruit specific bacteria in their rhizosphere, but if inter/intraspecific competition increases, is this rhizosphere recruitment affected? We found that this bacterial recruitment in the rhizosphere by alfalfa, brassica, and fescue is resilient. Additionally, that they are other beneficial bacteria being recruited only in the higher density plots, which we interpret as bacteria that may play a role in plant-plant co-existence. Lastly, there was only one example of possible sharing of rhizobacteria, where a bacterium typically associated with one plant had also established in the rhizosphere of another. In all, this study used rhizosphere samples to answer a new question.

The reviewer’s additional comments have also been addressed:

1. Reviewer #1: Title: The Shared Rhizosphere Bacteriome of Multiple Plant Species Reveals Species-Specific and Neighbor-Specific Recruitment. The title does not express the main conclusion of the work and is focused on part of the results. I would suggest one more comprehensive.

**We have changed the title to “Effect of intra- and inter-specific plant interactions on the rhizosphere microbiome of a single target plant at different densities”**

2. Abstract: Microcosm study containing different densities; have you measure the densities? or any previous report if you known this then please mention it.

**We did measure the densities by counting each plant individually. If this is regarding “low”, “medium”, and “high” terms used for our densities, they are just to reflect the relative density in this study.**

3. “Rhizobacterial beta diversity was promoted by increasing plant diversity around the target plant species. In contrast, rhizobacterial beta diversity was reduced by increasing plant density around the target plant species.” If possible please change this like Rhizobacterial beta diversity was promoted by increasing the plant diversity, whereas it was reduced by increasing plant density around the target plant species.

**We have made the recommended change.**

4. Add your significant finding which is missing? There is nothing wrong with the abstract. It is however quite limited in that value for the reader is not clear. How could the findings of this work be made more useful for the readers. Also, the significance of the findings seems to be low. Re-write the conclusions in abstract.

**We have slightly modified the abstract to highlight our findings. This study found that plants maintained their ability to recruit their specific bacteria despite escalating plant-plant competition. Moreover, deleterious bacteria were not identified in these highly competitive spaces.**

5. Material & Methods: Please indicate how many replicates per isolates/ treatment were tested, how many treatments were evaluated, how many time were the assays done, which controls were used, and include references for the methods. All this crucial information is missing.

**The number of treatments, replicates, and controls are listed in lines 94-96, 100-102, 104-105, 119-123, and 131-137. We have 17 citations in the methods.**

6. Table & Figure: Please, improve all the legends of the tables and the figures which are improper written. All figures need an upgrade in terms of design, and improved DPI with readable form of all names, etc. Please read this paper: Singh, R.K.; Singh, P.; Sharma, A.; Guo, D.‐J.; Upadhyay, S.K.; Song, Q.‐Q.; Verma, K.K.;Li, D.‐P.; Malviya, M.K.; Song, X.‐P.; et al. Unraveling Nitrogen Fixing Potential of Endophytic Diazotrophs of different Saccharum species for Sustainable Sugarcane Growth. Int. J. Mol. Sci. 2022, 23, 6242. https:// doi.org/10.3390/ijms23116242. The discussion is unable to guide the reader through a logical comparison of the results expressed and the current field of Rhizosphere Bacteriome of Multiple Plant Species Reveals Species-Specific and Neighbor-Specific Bacteriome. The authors should focus in the main results and discussed with actualized bibliography which is missed. Therefore, the manuscript needs to be improved after it can be considered for further publication. The authors need to revise the manuscript and the revised manuscript may contain additional data to justify the work.

**We have improved the legends for the tables and figures. Additionally, we have exported the figures directly to improve resolution of the figure. We thank the reviewer for recommending the article. We considered other methods of** **representing this data, much like the Venn Diagram in Figure 2. of the article which we have done in the past but decided to use the current differential abundance figure instead. We considered separating the upregulated and down regulated bacterial taxa but are unsure if that would be deemed as an improvement for studies the reviewer.**

Reviewer #2:

1. I initially found this manuscript interesting and clear, but ultimately, it left me disappointed. The paper aims to study the effects of plant neighborhood on the rhizosphere bacteriome of a primary plant, focusing on the density and diversity of neighboring plants. It investigates three species with different development patterns: Medicago sativa, Fescue spp., and Brassica juncea. While the study had the potential to provide valuable insights into the role of neighboring plants in the bacteriome of crop species, the experiment has already been published in: Newberger, D.R., Minas, I.S., Manter, D.K. et al. Shifts of the soil microbiome composition induced by plant–plant interactions under increasing cover crop densities and diversities. Sci Rep 13, 17150 (2023). <https://doi.org/10.1038/s41598-023-44104-8>. In this manuscript, another analysis is conducted using the same samples. I cannot identify sufficient new significant results or experiments compared to the previously published article.

**We are sorry to hear about this disappointment. In our cover letter to PLOS One we drew attention to this published article and included it in the manuscript submission, since we wanted to show that these were different studies. We would like to clarify that this experiment (the present submission) did not use the same samples as the Scientific Reports paper and answered a new question. The published article used plant biomass and bulk soil samples from a forage cover crop perspective, meanwhile the current study uses rhizosphere samples to look at plant recruitment of bacteria in general. The published article could not shed light on plant rhizobacterial recruitment, since it was not studied.**

2. Additionally statistical significance needs to be provided for the beta dispersion results shown in the tables, possibly by adding the standard deviation for each value. Without this information, the results cannot be interpreted correctly.

**Typically, studies just compare the dissimilarity and show if microbiomes are dissimilar or not. Here we show the beta dispersion value in a quantifiable value. We agree that the beta dispersion would be even more valuable with standard deviation but have instead run a Tukey's ‘Honest Significant Difference’ method (TukeyHSD).**

3. Out of the 12 replicates, only 5 were collected, supposedly discarding replicates that did not meet the density-diversity target. Were all the plants of the same species similar in size, or were there phenotypical differences? Comparing this with the bacteriome results would have been interesting. Later I found that plant biomass was covered in the previous paper, which added to my frustration.

Plants were not of the same size, and individual plants were smaller as plant competition increased.

**We are sorry to hear about your frustration. We have added those findings to the introduction of the manuscript. However, we disagree that for our question of rhizobacteria colonization, that plant biomass would have been interesting, since this was a below ground question instead of an above ground question.**

4. Were the plant seeds superficially sterilized? Clarification is needed, as it is later indicated that "unsterilized pregerminated seeds" were used. Wouldn’t it be better to pregerminate all seeds in vitro before preparing the pots? Other more specific and minor revisions: The methodology could be improved for clarity. Please include the statistical information in the figure captions.

**It is now clarified that initial seeds were unsterilized in Line 140. We are unsure if it would have been better to pregerminate all the seeds in vitro, since we wanted an even mix when the seeds were spread into the pots. If they were pregerminated, then randomly and evenly transplanting would have been challenging for that many plants.**

5. The tables could be improved for clarity by organizing them into four blocks: monoculture, each of the 2-mixtures, and the 3-mixture. This can be easily done with a wider line between blocks, which helps in locating the relevant data.

**We thank the reviewer for suggesting an alternate table format but disagree that it is an improvement. Below is what we believe you recommended as compared to what is in the manuscript:**

**Recommended:**

| **A**1 | **A**b2 | **B**a2 | **F**a2 | **A**bf3 |
| --- | --- | --- | --- | --- |
| 0.46 | 0.5 | 0.46 | 0.501 | 0.493 |
| **A**24 | **A**b24 | **B**a24 | **F**a24 | **A**bf24 |
| 0.458 | 0.46 | 0.424 | 0.488 | 0.494 |
| **A**48 | **A**b48 | **B**a48 | **F**a48 | **A**bf48 |
| 0.451 | 0.423 | 0.428 | 0.476 | 0.488 |
| **B**1 | **A**f2 | **B**f2 | **F**b2 | **B**af3 |
| 0.464 | 0.484 | 0.524 | 0.506 | 0.48 |
| **B**24 | **A**f24 | **B**f24 | **F**b24 | **B**af24 |
| 0.465 | 0.475 | 0.444 | 0.483 | 0.497 |
| **B**48 | **A**f48 | **B**f48 | **F**b48 | **B**af48 |
| 0.483 | 0.471 | 0.446 | 0.467 | 0.451 |
| **F**1 |  |  |  | **F**ab3 |
| 0.465 |  |  |  | 0.481 |
| **F**24 |  |  |  | **F**ab24 |
| 0.475 |  |  |  | 0.491 |
| **F**48 |  |  |  | **F**ab48 |
| 0.502 |  |  |  | 0.473 |

**Currently in the manuscript:**

| **A**1 | **A**24 | **A**48 | **A**b2 | **A**b24 | **A**b48 | **A**f2 | **A**f24 | **A**f48 | **A**bf3 | **A**bf24 | **A**bf48 |
| --- | --- | --- | --- | --- | --- | --- | --- | --- | --- | --- | --- |
| 0.460 | 0.458 | 0.451 | 0.500 | 0.460 | 0.423 | 0.484 | 0.475 | 0.471 | 0.493 | 0.494 | 0.488 |
| **B**1 | **B**24 | **B**48 | **B**a2 | **B**a24 | **B**a48 | **B**f2 | **B**f24 | **B**f48 | **B**af3 | **B**af24 | **B**af48 |
| 0.464 | 0.465 | 0.483 | 0.460 | 0.424 | 0.428 | 0.524 | 0.444 | 0.446 | 0.480 | 0.497 | 0.451 |
| **F**1 | **F**24 | **F**48 | **F**a2 | **F**a24 | **F**a48 | **F**b2 | **F**b24 | **F**b48 | **F**ab3 | **F**ab24 | **F**ab48 |
| 0.465 | 0.475 | 0.502 | 0.501 | 0.488 | 0.476 | 0.506 | 0.483 | 0.467 | 0.481 | 0.491 | 0.473 |

**We believe our table depicts the dataset in a better way.**

6. L37: Using both "change" and "can change" is redundant. Please remove one of them. L40: Remove the unnecessary "of".

**Thank you, this revision has been made.**

7. L105-106: Was the deionized water sterilized by filtration or another technique? L190: Deleting "and" would improve clarity.

**Thank you, this revision has been made and the water filtration method has been clarified.**

8. L127: Please provide more information on the sequenced reference soil (n=2).

**We have added where this reference soil was sampled from Agricultural Research, Development and Education Center-Colorado State University. It is used for internal quality control.**
